# Pollen preferences of stingless bees in the Amazon region and southern highlands of Ecuador by scanning electron microscopy and morphometry

**DOI:** 10.1371/journal.pone.0272580

**Published:** 2022-09-20

**Authors:** Joseline Sofía Ocaña-Cabrera, Jonathan Liria, Karla Vizuete, Cristina Cholota-Iza, Fernando Espinoza-Zurita, Claude Saegerman, Sarah Martin-Solano, Alexis Debut, Jorge Ron-Román

**Affiliations:** 1 Laboratorio de Biotecnología Animal, Carrera de Ingeniería en Biotecnología, Departamento de Ciencias de la Vida y de la Agricultura, Universidad de las Fuerzas Armadas ESPE, Sangolquí, Pichincha, Ecuador; 2 Grupo de Investigación en Sanidad Animal y Humana (GISAH), Carrera de Ingeniería en Biotecnología, Departamento de Ciencias de la Vida y de la Agricultura, Universidad de las Fuerzas Armadas ESPE, Sangolquí, Pichincha, Ecuador; 3 Research Unit of Epidemiology and Risk analysis applied to Veterinary Sciences (UREAR- ULg), Fundamental and Applied Research for Animal and Health (FARAH) Center, Department of Infections and Parasitic Diseases, Faculty of Veterinary Medicine, University of Liege, Liege, Province of Liège, Belgium; 4 Grupo de Investigación en Población y Ambiente, Universidad Regional Amazónica IKIAM, Tena, Napo, Ecuador; 5 Laboratorio de Caracterización de Nanomateriales, Centro de Nanociencia y Nanotecnología, Universidad de las Fuerzas Armadas ESPE, Sangolquí, Pichincha, Ecuador; 6 Grupo de Investigación en Sanidad Animal y Humana (GISAH), Carrera de Ingeniería Agropecuaria, Departamento de Ciencias de la Vida y de la Agricultura, Universidad de las Fuerzas Armadas ESPE, Sangolquí, Pichincha, Ecuador; University of Palermo, ITALY

## Abstract

Stingless bees are effective pollinators of native tropical flora. Their environmental service maintains flow of pollen through pollination, increase reproductive success and influence genetic structure in plants. The management of stingless bees “meliponiculture”, is an activity limited to the countryside in Ecuador. The lack of knowledge of their managers about pollen resources can affect the correct maintenance/production of nests. The objective is to identify botanical families and genera of pollen grains collected by stingless bees by morphological features and differentiate potential species using geometric morphometry. Thirty-six pot pollen samples were collected from three Ecuadorian provinces located in two climatically different zones. Pollen type identification was based on the Number, Position, Character system. Using morphological features, the families and genera were established. Morphometry landmarks were used to show variation for species differentiation. Abundance, diversity, similarity and dominance indices were established by counting pollen grains, as well as spatial distribution relationships by means of Poisson regression. Forty-six pollen types were determined in two study areas, classified into 27 families and 18 genera. In addition, it was possible to identify more than one species, classified within the same family and genus, thanks to morphometric analysis. 1148 ± 799 (max 4211; min 29) pollen grains were counting in average. The diversity showed a high richness, low dominance and similarity between pollen resources. Families Melastomataceae and Asteraceae, genera *Miconia* and *Bidens*, were found as the main pollen resources. The stingless bee of this study are mostly generalist as shown the interaction network. The results of the present survey showed that stingless bees do not collect pollen from a single species, although there is evidence of a predilection for certain plant families. The diversity indexes showed high richness but low uniformity in the abundance of each family identified. The results of the study are also meaningful to the meliponiculture sector as there is a need to improve management practices to preserve the biodiversity and the environment.

## Introduction

Stingless bees of the tribe Meliponini belong to the group of corbiculate bees as they possess an anatomical structure on the hind legs known as the pollen baskets [1] or “corbicula” [2] specialized in the storage and transport of pollen and resins [3] from plant to the nest. The main morphological characteristics are: reduction of the sting, little venation on forewings and reduction of the penicillum [2]. Based on the degree of speciation of these insects, South America is presumed to be the territory of origin and the starting point for their spread in Asia and Africa [4].

In Ecuador, the most recent study on native bees reported 132 species, classified in 23 genera, distributed in the 24 provinces of the country [5]. Being a megadiverse country, it is important to study meliponids to understand their ecological interactions and their importance in Ecuadorian forests.

The combination between the intertropical location and the altitudinal gradient caused by the Andes mountain range creates a diversity of habitats that give rise to distinct floristic elements [6]. The number of vascular plant species in Ecuador exceeds 17,000 [7]. Over the last few years, harmful environmental effects have been observed in wild bees, leading to a decrease in the plant-pollinator ratio and consequently, with a decrease in plant diversity [8]. Agriculture, mechanical and chemical treatments are examples of activities that has affected the natural habitat of wild bees [9]. In 2014, FAO proposed topics on which efforts should be focused due to the lack of information on pollination as an environmental service, especially in Latin America and the Caribbean [10].

Stingless bees build their nests in various cavities (abandoned bird nests, cement blocks or under the ground) [11], with different nesting features such as the shape of the nest entrances or protection of the brood discs (exposed or protected by a layer of wax or involucre) [12]. The nest is protected and divided by batumen, a composition of mud, faeces, resins, or plant material such as seeds, mixed or not with wax [13]. Internally, nests are made of cerumen a construction material composed of a mixture of wax, resins and other sticky substances of plant origin [14, 15]. It also serves as an antimicrobial [16] and antifungal [17] protection. Several layers of cerumen form the involucre, a structure that surrounds, protects and maintains temperature [13, 18, 19], in the brood chamber of the nest. Nest food storage is in pots made of soft cerumen that are located outside the involucre, round and small for honey pots, large and elongated for pollen pots [20].

In the tropical zone of America more than 500 species [1] of stingless bees plays an important role as pollinators in maintaining the forest diversity [21] ensuring its reproduction thanks to the dispersion of pollen [22]. Moreover, stingless bees also maintain the stability of economically important crops in agroforestry projects [23, 24] and help to reestablish the ecological balance in invaded endemic areas [25, 26].

Stingless bees meet the need for pollination of endemic plants, thanks to their species diversity and adaptive capacity [27]. For instance, the genera *Bombus*, *Centris*, *Eulaema*, *Melipona* and *Cylocopa* use a specific mechanism for pollen collection, their thoracic muscles vibrate inside the flower releasing pollen that sticks to the body [28]. Native bees visit flowers that *Apis mellifera* cannot because of their larger size compared to *Melipona* species [29]. Stingless bees visit between 15–20% of angiosperm plants [30] their pollination services cover between 30,000–50,000 species [31]. In developing countries, meliponiculture, manage and care of stingless bees, continues to be an informal economic activity, or even considered a hobby because there is not much scientific knowledge or standardized processes about it [32].

The health status of the stingless bee colonies is dependent on the great diversity of plants from which they obtain resources [33]. Their foraging activity depends on the floral availability of the environment in which they live, which is specialized temporarily when a certain plant species offers attractive resources [34]. Pollination service provided by bees increases reproductive success in plants, influences the genetic structure of populations and plays a major role at the evolutionary level [9, 35, 36]. In the specific case of *Apis mellifera*, its floral constancy promotes geitonogamy and reduction of genetic exchange [37]. It is important to know if this behavior also occurs in stingless bees or if their role as pollinators maintains biodiversity.

Pollen, as the object of study, is the male gamete of phanerogamous plants, formed by a vegetative cell that has sperm cells enclosed in a cytoplasm surrounded by an external wall or exine [38]. Pollen is the main source of protein and carbohydrates for stingless bee brood, adult bees and queens [33]. It is used inside the nest for the production of honey and bee bread [39]. At palynological level, the national databases lack sufficient information but it exists a database in constantly update that includes some Ecuadorian plant information [40]. As a guide in this survey a compilation of information about botanical families used by stingless bees was developed (**S1 Table**) [41–91].

Pollen origin analysis are generally conducted by light microscopy techniques [92]. Distinguishing pollen characteristics requires exceptional instruments, for instance, a Scanning Electron Microscope (SEM), that allows through microphotography to describe shape and morphological size characteristics of pollen to classify it into a family, a genus or even a species level [93, 94]. The lack of information on pollen description and identification is related to the lack of information on bee-plant interactions and food source plants in Ecuador. It makes it necessary to generate scientific data that will benefit meliponicultors (managers of stingless bees).

The objectives of the survey were: i) to identify the families and genera of plants used as food sources for stingless bees in three provinces of Ecuador through morphological analysis of pollen grains (pg) collected from meliponid nest pots. This allowed us to ii) determine the preference of native bees for certain Ecuadorian plant families in two climatologically different areas, as a tool for species conservation and to contribute to the knowledge of meliponicultors.

## Materials and methods

### Study design and sampling

**Fig 1:**
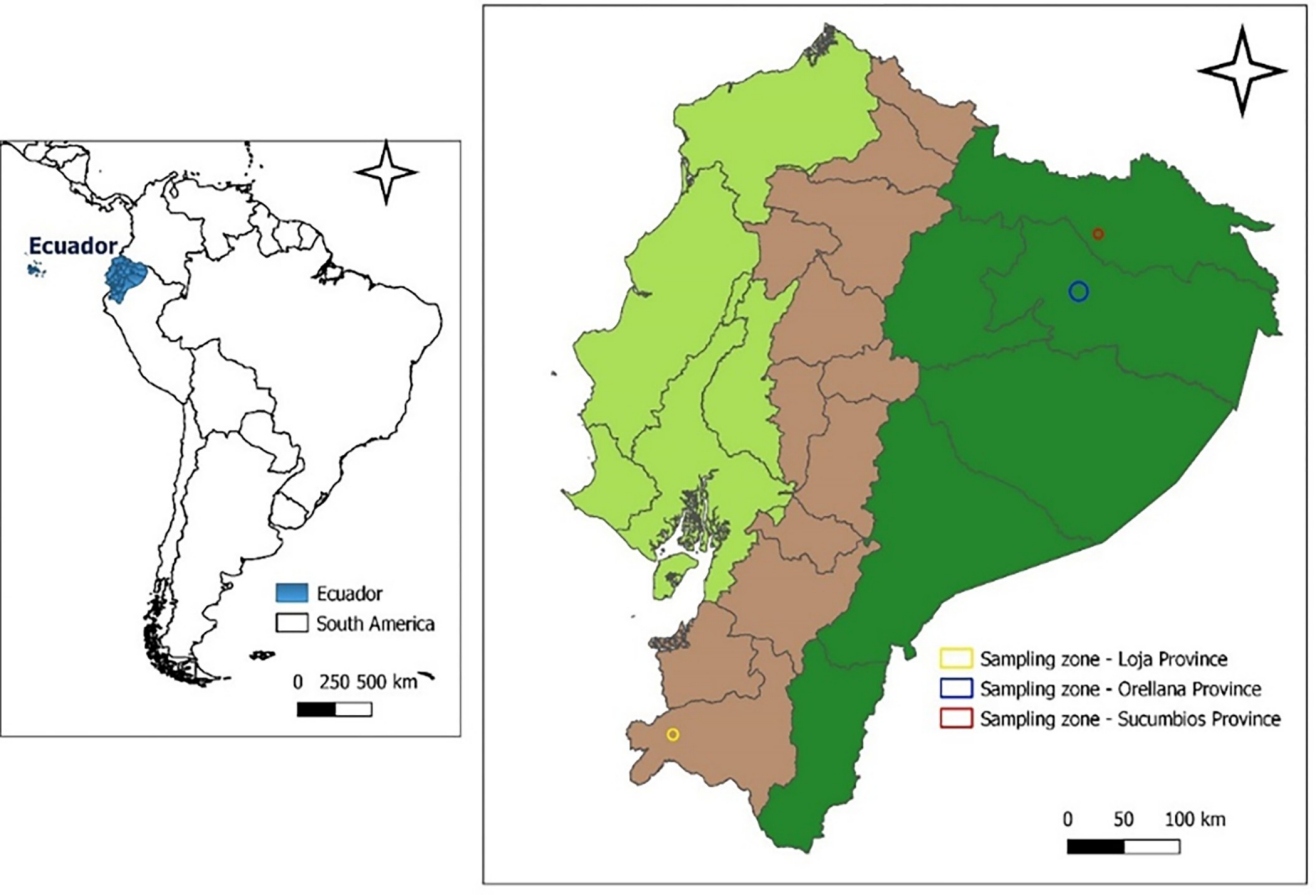
Geographical location of the study areas, in three provinces of Ecuador. Layers were obtained from: http://www.efrainmaps.es. Carlos Efraín Porto Tapiquén. Geography, GIS and Digital Cartography. Valencia, Spain, 2020 for America layer, Instituto Geográfico Militar, 2008, Base nacional escala 1:1’00.000 for Ecuador layer.

In this cross-sectional survey, two provinces of the Amazon region (Orellana and Sucumbios) and one province of the southern highlands region (Loja) of Ecuador were defined as sampling regions (Fig 1) (Table 1). During field work, small scale meliponicultors were contacted in the three areas. In absence of a sampling frame, the field visits were guided by a local field technician with expertise in Meliponiculture. Meliponicultors were given an informative survey and signed a consent for the collection of pollen samples from their nests in April, August-September and December 2018.

**Table 1:** Geographical location and number of the sampling zones.

Ecuadorian Region	Province	Location	Coordinates (UTM)^a^	Altitude (m.a.s.l.)^b^	Number of nests	Number of samples
Amazon	Sucumbios	Shushufindi	0°11’14”S^c^ 76°38’42”W^d^	>262	4	6
Amazon	Orellana	Taracoa	0°26’08”S^c^ 76°46’20”W^d^	>269	10	10
		Dayuma	0°40’14”S^c^ 76°52’54”W^d^	>275	7	10
Southern highlands	Loja	Pindal	4°06’51”S^c^ 80°06’28”W^d^	>801	10	10

^a^ Universal Transversal Mercator coordinates system

^b^ Meters above sea level

^c^ South

^d^ West.

### Sample collection

**Fig 2:**
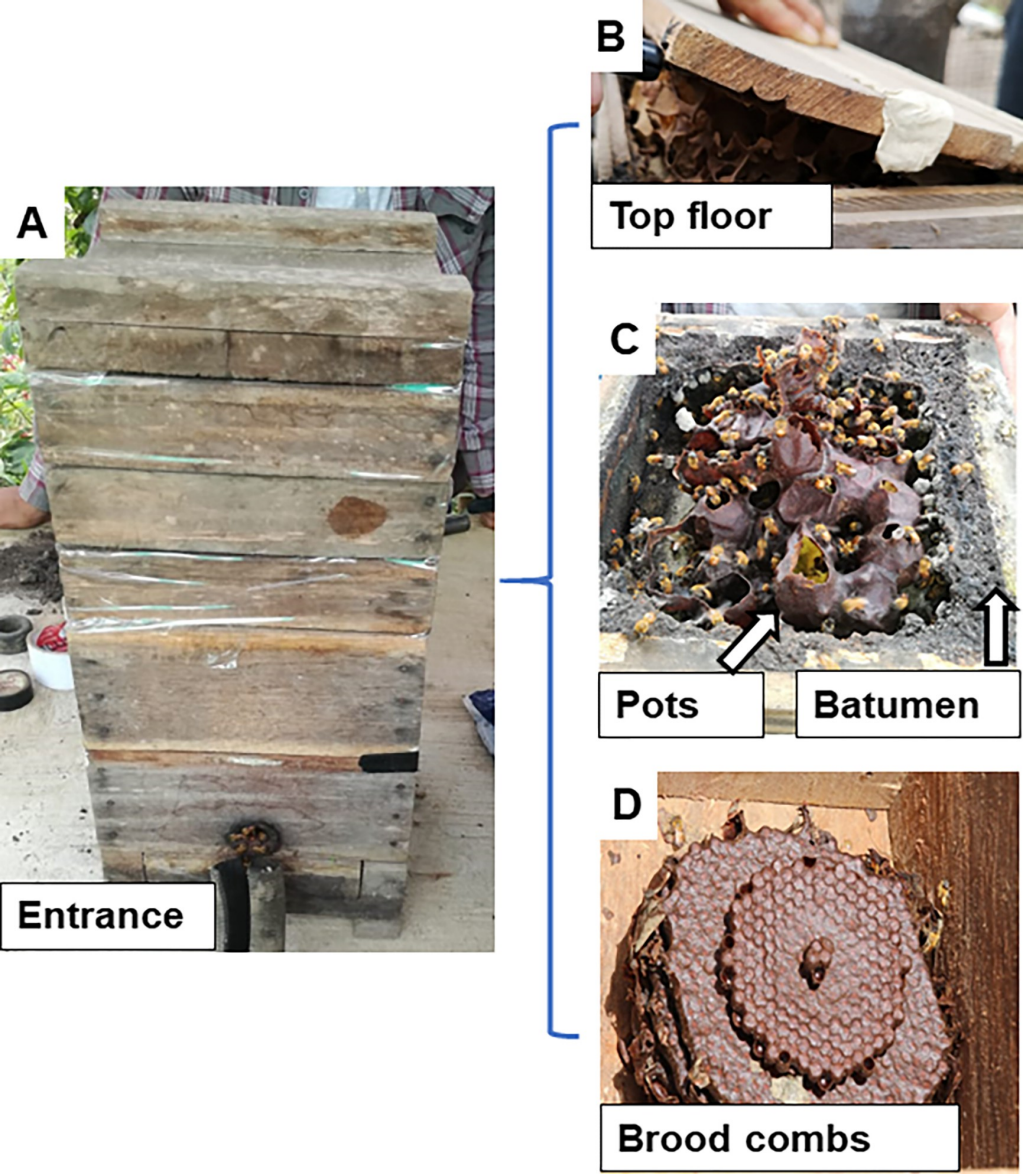
Technical nest of stingless bees in Ecuador. A) The nest consists in three main zones. B) top floor is the way by meliponicultor can open the nest and managed the bees. C) The storage zone cover by batumen (material made from resins and mug) contains honey and pollen pots. D) The brood chamber where different “plates or discs” are stacked in a column. *Note*: a natural nest has the same structure but inside a tree log.

Pollen grains stored in pots out of stingless bee nests were collected during the transfer from natural nests to a technical nest (Fig 2) by taking the pollen directly from the top floor (storage place of stingless bee pollen pots) of the box or technical nest. The selection of one pollen pot by nest was random. Only sealed pots were sampled. Three to six grams of pollen were extracted from the pot with the help of a sterilized paddle; one paddle was used for each pot. The samples were stored at 4°C in sterilized tubes, labelled with the following codes: H as the number of the meliponary, N as the number of the nest and then sealed until their transfer to the Animal Biotechnology laboratory at the Forces Armed University ESPE, Sangolquí, Ecuador.

Pollen as a product of a stingless bee nest is classified as a food supplement and/or medicinal product. The objective of collection was socialized with each owner, them approval was obtained by reading and signing an informed consent form.

### Acetolysis and cleaning of pollen grains

Protocol of acetolysis [95] was used with modifications. To 0.1 g of pollen, 600 μL of washing solution was added composed of glycerol and hot distilled water (1:5). Solutions was then centrifuged at 2500 rpm for 10 min. The supernatant was discarded, and another wash was performed with 600 μL of hot distilled water; it was then centrifuged (250 rpm, 5 min) and the supernatant was discarded. To separate the sediment, 200 μL of warm distilled water was added, centrifuged (2500 rpm, 5 min) and the supernatant was removed and dried at room temperature (i.e., 20°C) for 10 min in a laminar flow cabinet. 1 mL of acetolysis solution (sulfuric acid: glacial acetic acid, 1:9) was added and placed in a water bath at 70°C for 20 min; centrifuged (2500 rpm, 10 min) and the supernatant was discarded.

Three drops of tween 20 plus 200 μL of warm distilled water were added, strongly vortexed and centrifuged (2500 rpm, 15 min). Subsequently, bacteria were eliminated using 12.5 μL of the Streptomycin antibiotic (20 μg/μL) and distilled water was added to complete 250 μL to wash the pollen grains. It was left in contact for 24 h, centrifuged (3500 rpm, 15 min) and the supernatant was removed. Samples were stored refrigerated until lyophilization.

### Preparation and observation in scanning electron microscopy

The drying process by lyophilization was carried out in the equipment (ILSHIN, model FD 5508) for 24 h, at -62°C and 1.2 Pa; samples that were placed in the equipment were previously frozen liquid nitrogen for 3 min. Once dry, the sample was dispersed on a copper foil glued to the aluminum sample holder. Sample was covered with a 20 nm layer of gold before being analyzed by SEM (TESCAN, model MIRA 3, field emission gun).

**Fig 3:**
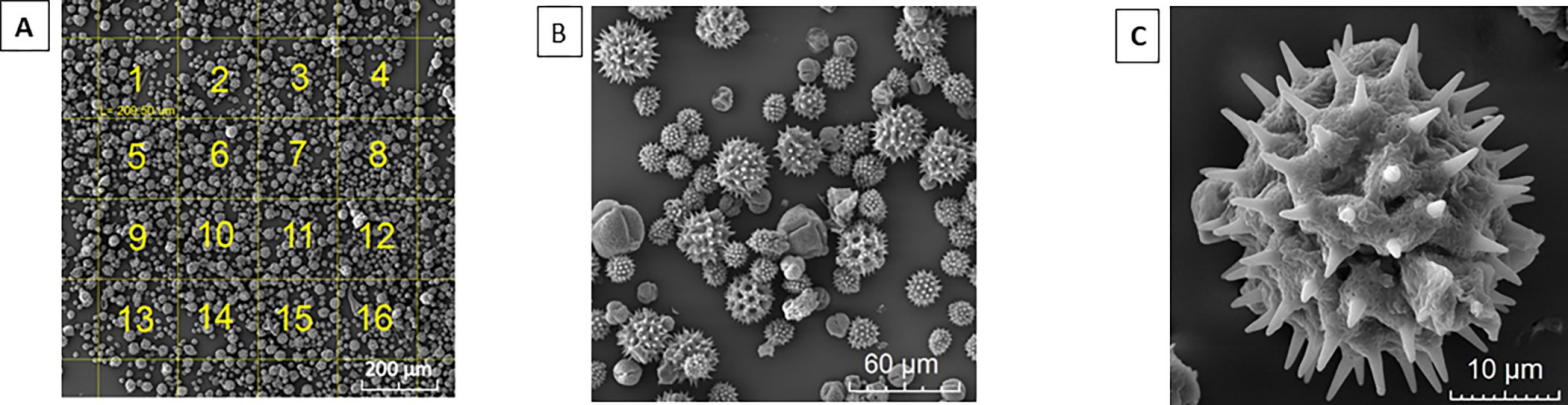
Scanning electron microscopy methodology. A) Area with homogeneous dispersion, 1 x 1mm. 200X; B) Counting quadrant 990X; C) Single photograph, magnification depending on pollen grain size (1.6–15 kx).

To count and identify pollen, microphotographies were taken, identifying an area of approximatively of 1 mm2 with uniform dispersion of pollen grains (at 200 x magnification). Each area was then divided into 16 quadrants (at 990 x magnification) for the respective count. Then, each pollen grain was photographed individually, with a magnification between 1600 and 15000 x, a parameter that was adjusted according to the size of each one (Fig 3).

### Counting and morphological description

In each quadrat, pollen types were counted and differentiated manually, identifying each one with colors. To establish the abundance percentages [96] was considered. Pollen grains with abundance ≥ 10% were considered "really important" food sources. Pollen grains with abundance ≥ 90% are considered pollen sources of "temporal specialization" [97]. The following measurements were obtained from the individual photographs: polar and equatorial diameter (μm), P/E (polar diameter/equatorial diameter ratio), area (μm^2^) and perimeter (μm), by means of which size and shape could be defined [98].

Thanks to the magnification achieved with SEM, the characteristics of the exine, openings and ornamental elements of the pollen wall were identified, and using the NPC system (number, position of openings, and characteristic of the exine) [99], pollen grains were classified into pollen types.

### Classification into family and genus

To classify pollen grains into family and/or genus, morphological descriptors and pollen type were used. For this purpose, scientific publications [100–103], books [12, 104, 105] and pollen databases were consulted: National Centre for Environmental Information [106], Apibotanica [107], the Global Pollen Project [108], Oreme [109], and mainly from Latin America [110]; to perform a comparison of measurements and shapes.

### Morphometric analysis

**Fig 4:**
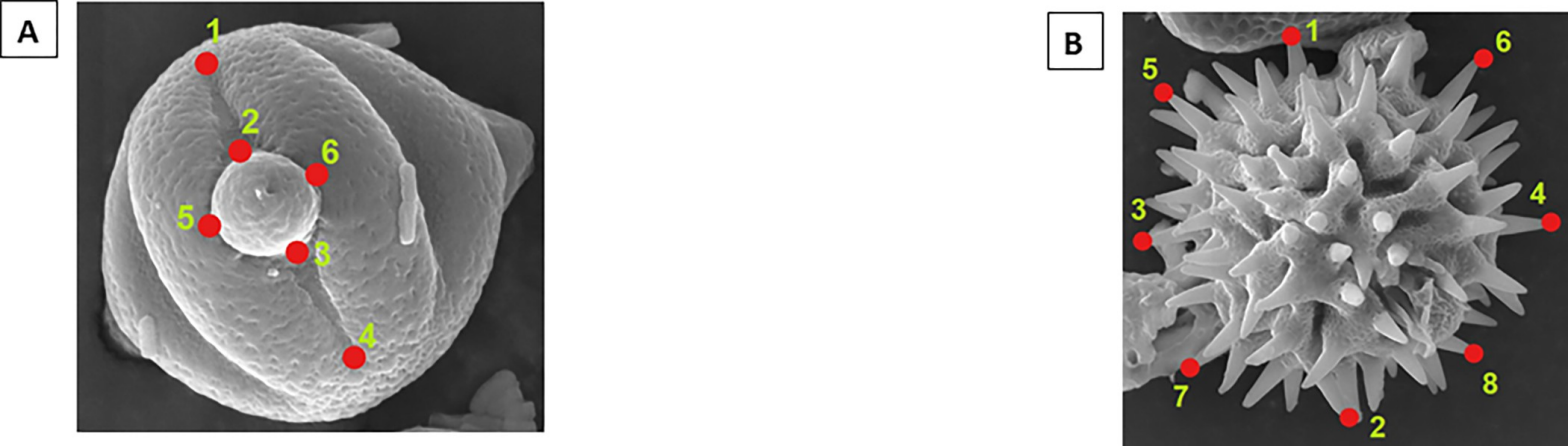
Morphometric geometry methodology. A) Selected anatomical points for pollen from family Melastomataceae, genus *Miconia*; B) Selected anatomical points for pollen from family Asteraceae, genus *Bidens*. In the figures above each red point represent the specific position (coordinates) of one landmark or anatomical point, that surrounding the area which were used as potential indicator of species differentiation for pollen grains that belongs to the same family and genus.

Sections of the pollen grains that showed the greatest morphological variation were selected and these areas were marked with anatomic points or landmarks. Three photographs were used for each pollen grain, from which x, y coordinates were digitized using the TPSDIG program [111]. Conformational differences were analyzed in MorphoJ software, stated by [112] using Principal Component Analysis (PCA) as an exploratory technique to visualize the axes of greatest variation, and then Canonical Variables Analysis (CVA) to evaluate significant differences between the different groups (taxonomic categories) [113]. The pollen grains that were processed belong to the following families: Melastomataceae and Asteraceae. For the first family, six landmarks were selected (Fig 4A) in the equatorial zone that provided differential data based on the presence or absence of a pore and the width of the aperture. For the second family, eight landmarks (Fig 4B) were selected that differentiated the length of diameters and structures known as spicules.

An example of pollen grains that did not enter this analysis are the families Molluginacea and Piperacea, since the pollen grains are apolar and inapertured, being absent characteristics that allow their differentiation by means of landmarks.

### Distribution of pollen grains

In this study the Poisson distribution was used, which describes the probability with which an event can occur during a certain interval, whether of time, distance, area or volume [114]. The random variable of the distribution was the number of times that a certain pollen type appeared in the area (1 mm^2^) of the SEM stub. R Commander software was used to verify that pollen grains counting follows Poisson distribution. In addition, Poisson Regression was also used to determine the spatial distribution of the families’ plants found. The dependent variable was the number of pollen grains per pollen type (independent variable).

### Calculation of biodiversity indexes

Based on pollen grains counting and the identification of families according to morphology, the Shannon index was calculated. It quantifies the specific biodiversity, that is, the non-uniformity of an area, taking into consideration the total number of families identified and the abundance of each one [115]. Simpson’s index was also calculated, which represents the probability of randomly taking two individuals from a population and having them belong to the same species; it is an estimator of the dominance of certain plant species in a given area [116].

Finally, the Jaccard index was used to measure the degree of similarity in terms of families used as pollen resources inside the pot by nest, this index is a type of inverse measure to the diversity estimated by Shannon [117].

### Network interactions

It was possible to establish preference relationships based on pollen grain counting between stingless bees and plant families. Stingless bee genus and species were identified in two parallel studies from this, using entomology [118] and molecular biology techniques for the identification of native bees by [119]. R Studio software was used to obtain the networks and the program developed by Dr. Dáttilo Wesley, PhD, of the Instituto de Ecología, A.C., Mexico [120].

## Results

As explained in the methodology, the pollen grains (pg) that were completely analyzed were those that, during the initial count, exceeded 10% abundance in the selected stub area (i.e., important food sources). Thereafter, new percentages were established to provide an order, according to the study area.

The collection of 36 pollen samples from the three provinces located in the Amazon region and Southern highlands of Ecuador resulted in 2433 micro photographs, from which 54 pollen types (6533 pg) were identified in Sucumbíos, 84 pollen types (23579 pg) in Orellana and 49 pollen types (11232 pg) in Loja. This count made it possible to establish abundance percentages (Table 2) and to relate similar pollen types between provinces, identifying a total of 46 pollen types and 28 botanical families. Only 14 of the 28 families found could be classified "genus" at generic level, identifying 18 (Table 2), thanks to the morphological description of the different pollen types and the ornamentation of the exine (S2 Table).

**Table 2:** Families, genera and their abundances of pollen grains identified by province.

		Abundances by province		
Family	Genus	Sucumbíos	Orellana	Loja
Alismataceae			+	+
Aizocaceae				+
Anacardaceae			+	
Arecaceae				+
Asteraceae	*Ageratum*			+
Asteraceae	*Bidens*		+	++
Asteraceae	*Iva*			+
Berberidaceae			+	
Bursecaceae	*Bursera*	+		
Bursecaceae	*Quercus*		+	
Cyperaceae	*Cyperus*		+	
Cytinaceae			+	
Euphorbiaceae			+	
Fabaceae	*Chamaecrista*		+	+
Fabaceae	*Inga*		+	
Lardizabalaceae			+	
Lythraceae	*Hemia*		+	
Loranthaceae		+		
Melastomataceae	*Miconia*	++++	++	+
Molluginaceae	*Mollugo*	+		
Molluginaceae			+	
Oleaceae	*Fraxinus*		+	
Papaveraceae			+	
Piperaceae			+	+
Plantaginaceae				+
Plumbaginaceae	*Plumbago*	+		
Poaceae			+	
Polygalaceae	*Polygala*			+
Rosaceae	*Prunus*	+		
Salisaceae	*Salix*	+		
Sapindaceae	*Paullinia*		+	
Vitaceae	*Cissus*	+		

Crosses represent 0–25% (+), 26–50% (++), 51–75% (+++), 76–100% (++++).

Regarding morphometric analysis to identify species, significant differences were determined between pollen grains belonging to *Miconia* (Melastomataceae) at province level, between Loja—Sucumbios (discriminant function between Mahalanobis distance, p-value < 0.0001) and between Orellana—Sucumbios (p-value < 0.001); which means that there is more than one species of plants of the genus *Miconia* used as a pollen resource by stingless bees of these provinces. However, between the provinces of Orellana and Loja, there is no difference between the pollen grains (p-value < 0.08), which suggests that possibly, pollen grains belonging to the same species of *Miconia* were selected for analysis or that the area of the pollen grain does not act a discriminating parameter between plant species of these two provinces.

Pollen grains of *Bidens* (Asteraceae) were identified as resources only in the provinces of Orellana and Loja. There was a significant difference (discriminant function between Mahalanobis distance, p-value < 0.008) between those belonging to each province. It means that there is at least one species of *Bidens* collected by native bees. A significant difference was observed even among meliponaries of each province (0.005 < p-value < 0.03), i.e., there are plants of several species of *Bidens*.

**Fig 5:**
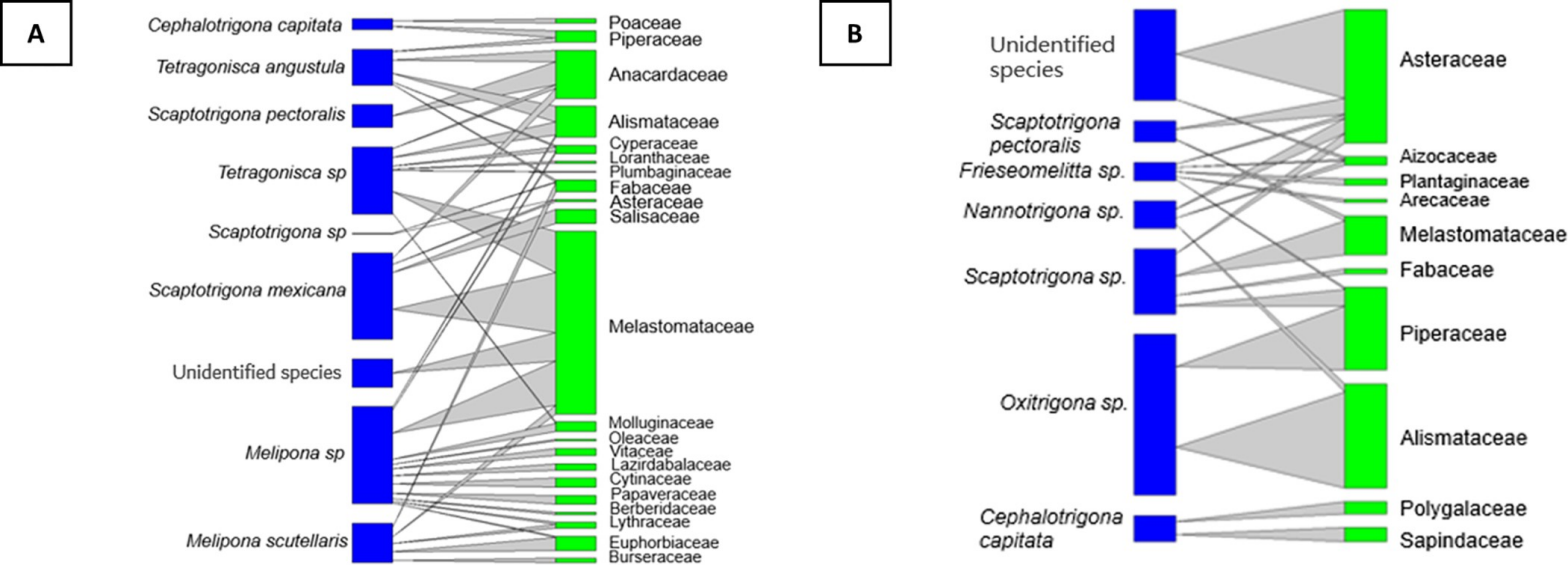
Bee-plant interaction network. **A)** Amazon region. **B)** Sierra South region.

As shown in Table 2, the only botanical family that was present in the three study areas was Melastomatacea, which is why it was decided to choose this family as an independent variable in the spatial distribution analysis using Poisson regression. It was verified that the pollen of this family is mostly used (< 70% of Sucumbios nests and < 30% of Dayuma nests) as a food resource (p-value < 2e-16) by stingless bees in the Amazon region. It was also verified that the collection of pollen grains of the Melastomataceae family is significantly related to the collection or storage of pollen of Burseraceae, Euphorbiaceae, Fabaceae and Molluginaceae (2e-16 < p-value < 0.001). In addition, interaction networks between the identified bee species and the families used as food resources were carried out, determining that *Melipona*, *Tetragonisca* and *Scaptotrigona* are the most generalist species of the Amazon Region (p-value < 0.001), as shown in Fig 5A. It is important to say that pollen grains of the Molluginaceae play an important role as a food resource (p-value < 0.001).

Asteraceae was the next family to have significance after the statistical analysis with Poisson regression, identifying that Asteraceae is the most important food resource for stingless bee nests in Pindal, Loja (p-value < 0.001). Moreover, if we compare with the rest of the families, if stingless bees collect pollen from Asteracea family plant is highly probably that also recollect pollen from Melastomataceae and Aizocaceae (p-value < 0.001) due to the floral spatial distribution within each meliponary. Regarding the interaction with bee species, it was obtained that *Nannotrigona* and *Frisiomelita* are closely related to the collection of Asteraceae pollen grains (both are small species) (p-value < 0.001), and *Frisiomelita* is the most generalist in Pindal—Sierra (p-value < 0.001) (Fig 5B).

The calculation of the diversity indexes (Shannon’s index, H’) of families identified per sample, resulted in low values, for Sucumbíos: 0.97 > H’ > 1.72, for Orellana 1.86 > H’ > 2.17, for Loja 2.22 > H’ > 2.34. The data of the diversity indexes at sample level follow a normal distribution (Shapiro-Wilk, p-value = 0.12). There are no significant differences between the diversity indexes of the three zones (Kruskal-Wallis, p-value = 0.29).

There was no dominance (Simpson’s index, λ) of a single family in any study area, with values of 0.23 > λ > 0.39 for Sucumbíos, 0.18 > λ > 0.22 for Orellana, λ = 0.17 for Loja. The similarity (Jaccard index, JI) between plant families found in the three study areas was low, with values between 0 > JI > 0.25. However, when comparing families found in provinces of the Amazon region, Sucumbíos and Loja, these presented a higher similarity index: 0 > JI > 0.43. However, this similarity was strongly higher between Orellana and Loja: 0 > JI > 0.66, provinces of different regions and climatic zones of the country (see S3 Table).

## Discussion

The objective of the study was to identify the main families and genera of plants used as food sources by the stingless bees in two climatologically different regions of Ecuador. Twenty-eight families were found and identified into 19 genera. The existence of more than one species of the same genus was discriminated using geometric morphometry. Preference relationships were established between botanical family and stingless bee species. Values of diversity, dominance and similarity indexes were defined using pollen grain counts. Taxonomic classification was performed based on morphology and measurement of morphological parameters using microphotographs of pollen. In this sense, it is well known that exine shape and the generation of patterns on it, which are characterized by their high taxonomic specificity, are due to several protein subunits that are synthesized in the paternal myocyte and cellular interactions [130]. The shape of pollen grains and size, are used to reach at precise taxonomic identification, since generally, those pollen grains belonging to the same species have this constant characteristic, which is related to the chromosomal number of each plant [131]. Pollen can present a continuous wall or not, depending on the treatment given prior to microscopic observation. It can also expands when hydrated and the proximal poles (equatorial) fold if subjected to drying treatments such as acetolysis or natural fossilization processes [132]. On the other hand, the divergence in the wall and the variation in the measurements of the morphological parameters, possibly indicate a hybridization in the origin of the plant or the influence of the habitat in which it has developed [133].

Melastomataceae was presented as the only family in common for the three provinces of study, its pollen generally has apertures that classify it as psyllulate or rugulate and a heterocolpate or stephanocolpate conformation, characteristics that facilitate its existence in diverse climatological habitats [99]. In Brazilian forests, this family is typical as a source of pollen for stingless bees, since coevolution with bees of the genus *Melipona* has been verified [134]. Asteraceae occurred in two provinces, one in each region of the country. This family has the second-largest representation in Ecuador and its pollen is characterised by having spines or hooks on the exine [135], which facilitate its adhesion to the body of pollinators, thus ensuring its dispersal to other plants [131]. Molluginaceae previously reported in the Galapagos region [136] has pantocolpate pollen grains and spinulose exine [73] is considered one of the three families with the greatest preference for female bees in southern Africa [137].

Morphometric results of Melastomataceae, show the possible existence of more than two species belonging to the genus *Miconia*. However, it was not possible to identify the species since it is necessary a phenological study that relates the pollen of the nests with the pollen taken directly from plants. In terms of evolution, *Miconia* is known to be monophyletic in origin, and of the total number of species within this family, 25% have this origin [138]. *Miconia* is distributed throughout Latin America and have points of diversity in the Andes and in the humid forests of Brazil, which is why it is considered a genus of basic plants in all phytogeographic formations [139]. In Ecuador, these plants are used for medicinal, ornamental, food and construction purposes [140], but there is little information on their role as a pollen or nectary resource for stingless bees. The ecological importance lies in their high repopulation capacity in ecosystems altered by human intervention [141]. A degraded terrestrial ecosystem is recognized when there has been partial or total depletion of plants and soil nutrients [142]. Different studies on ecological restoration in southern provinces of the country caused by intensive cattle grazing, demonstrated the important role of *Miconia* plants in soil restoration, because it was observed growing after 2 years of total disappearance of plants and a great abundance after 10 years. This is mainly due because *Miconia* plants are good water catchers, maintaining soil moisture and fertility [143]. A common pattern of the sampled areas is the number of areas intervened by human, and in this study, it is evident how native bees have taken advantage of this natural ecological restoration, to obtain food resources and carry out, indirectly, the pollination that is supposed to have contributed to the restoration of these areas. Additionally, bees of the genus *Euglossa* temporarily specialize in collecting pollen from *Miconia chamissois* as a food source for their offspring [144].

The morphometric results for Asteraceae indicate significant differences between the pollen grains of *Bidens*, suggesting that there are also more than two species [145]. Plants of *Bidens* are used as forage for domestic animals, since their growth occurs in areas with abundant organic matter, near rivers or stream channels [146]. They are found in disturbed soils and are characterized by abundant nectar production during the summer season making them a very good source for bees [147].

Molluginacea and Piperacea pollen grains are unapertured and apolar [148]. According to the data and observations obtained, it is possible to raise hypotheses. The first one is that stingless bees classify pollen grains according to the species of origin and the second one, the pollen grains found in each pot may well be indicators of the spatial distribution pattern of plant species surrounding the nest. If a continuous habitable space is defined, it is possible to affirm that a population follows random, uniform or aggregate distribution patterns [149]. Investigations of this spatial dispersion allow the identification of inter-and intraspecific coexistence relationships, in addition to knowing the diversity of the ecosystem under study [150].

In this sense, there are families that presented significant degrees of relationship, Melastomataceae ~ Burseraceae, Euphorbiaceae, Fabaceae, Molluginaceae and Asteraceae ~ Melastomataceae, Aizocaceae, are possibly randomly distributed in the habitat outside the nest. The random distribution explains that each point in the environment is occupied by an individual and its presence does not affect the presence of another [151]. Pattern distribution of the rest of the families identified and that did not present a significant relationship, probably comply with a uniform distribution pattern, which is characterized by presenting a negative interaction expressed as competition for resources [152]. Melastomataceae and Asteraceae presenting an important significance in this study, probably follow an aggregate distribution in the space occupied by the bee nests, i.e., there is probably a positive attraction between individuals, at species level, since their distribution occurs by the formation of dense groups [151].

The existence of stingless bees and meliponiculture as such influence the maintenance of biodiversity thanks to the division and conservation of colonies or nests of these insects [152]. Stingless bees are the main pollinators and floral visitors of tropical native plants [47]. A complete specialization of bees towards plants is unlikely and is demonstrated in this study; stingless bees do not have a specialization towards certain plant [153–155], in addition the food source plants do not bloom throughout the year and therefore the same food source cannot supply the needs of the nest or colonies that have a perennial character [131, 156].

Abundance percentages reflect an order of preference [157–159]. Different authors have suggested the preference of stingless bees for specific plants according to their inflorescences. Such constancy is associated with the effectiveness of these insects as pollinators, since it facilitates both the harvest and the deposit of pollen grains, and ensures the reduction of contamination of stigmas with other pollen, optimizing the travels of worker bees to obtain nectar, pollen and resins [160]. Biologically, their polyilecty and great adaptability allow them to pollinate both endemic and introduced plants [47].

In Ecuador there are a lack of databases that can be used as starting point or loading information to contribute the information around the country. The potential of bees as bioindicators of the health environment is well known within biomonitoring, due to their foraging job [161]. Bees can storage in their nest large quantities of soil, vegetation, air and water residuals [162]. These characteristics turn bees in effective monitoring agents of zones affected by human practices.

## Conclusions

The results of the present survey showed that stingless bees do not collect pollen from a single plant species, although there is evidence of a predilection for certain plant families. Forty-six pollen types were reported that complied with the characteristic of having abundance higher than 10%, i.e., a real food source, in the three study zones, which were classified into six families and seven genera for the province of Sucumbios during April, 19 families (14 genera) in Orellana between the months August—December; and 10 families (8 genera) in Loja in September, in the year 2018. *Miconia* (Melastomataceae) was presented as the main source of pollen as it was found in the three study areas, followed by the families Asteraceae, Alismataceae, Piperaceae, Fabaceae, Bursecaceae and Molluginaceae.

This survey is the first to shows the importance of morphometric analysis as a support in the differentiation of taxa between provinces and meliponaries for pollen grains of the Melastomataceae and Asteraceae. These results suggest the existence of more than one species, belonging to the same genus, to both *Miconia* and *Bidens*.

The diversity indexes showed high richness but low uniformity in the abundance of each family identified. Calculations was made using each pot as the calculation unit. This suggest that inside of each pot, stingless bees storage just one type of pollen whose classification depend on floral origin. This last sentence could be a theorical explanation.

Stingless bees could be used as a sentinel, to show the health of the environment, specifically in tropical zones. In Ecuador study areas were close to oil exploitation, cattle farms and crops, therefore the possibility of finding traces of chemicals substances is a potential indicator of contamination.

As a recommendation, the usefulness of developing software (artificial intelligence) as a computer tool that allows the classification of pollen grains from microscopy photos is highlighted. It is also recommended to: i) use molecular biology techniques to support certain concepts about the biology and ecological role of stingless bees; ii) carry out the identification of plant species, using pollen, through a phenological study in the study areas of the nests, in order to compare findings by pollen and botanical identification.

## Supporting information

S1 Table Botanical families review chart. Plant families features used as pollen and nectar resources by stingless bees [41–91, 126].
(DOCX)

S2 Table Morphological description, and their parameters, of pollen grains with the highest representation in the three study areas [43, 47, 52, 84, 86, 99, 100, 118–129]. (DOCX)

S3 Table Indexes explaining chart. Comparison between the diversity, dominance and similarity indices in each study area.
(DOCX)
