# Supplementary material for: Pollen preferences of stingless bees in the Amazon region and southern highlands of Ecuador by scanning electron microscopy and morphometry
Source: PLoS One. 2022 Sep 20;17(9):e0272580. doi: 10.1371/journal.pone.0272580 (PMC9488792; doi:10.1371/journal.pone.0272580)
Supplement: S1 Table — Plant families features used as pollen and nectar resources by stingless bees [41–91, 126]. (DOCX) [file pone.0272580.s001.docx]

**S1 Table. Botanical families review chart.** Plant families features used as pollen and nectar resources by stingless bees.

| **Family** | **Geographical distribution** | **Resource used** | **References** |
| --- | --- | --- | --- |
| Aizocaceae | Dry parts of tropical and subtropical zones. Surroundings of Africa, Mediterranean, Asia Minor and the western coast of America (North, South and Caribbean). | Pollen and nectar | [41]; [42]; [43] |
| Alismataceae | Zonas tropicales de los 5 continentes (pantropical), zonas de clima templado y neotropicales | Pollen and nectar | [44]; [45]; [46] |
| Arecaceae | Tropics and subtropics of both hemispheres, extending to warmer regions | Pollen | [47]; [48]; [49] |
| Anacardaceae | Across tropical and subtropical zones | Pollen, nectar and resins | [50]; [51]; [52] |
| Asteraceae | Every environment and continent, except Antarctica. | Pollen and nectar | [43]; [53]; [54] |
| Berberidaceae | Mesophytic forests (average humidity and temperature) of Asia and America. | Pollen | [52]; [55]; [56] |
| Burseraceae | Paleotropical zones of South America, continental Asia and Africa. | Pollen | [57]; [58] |
| Cyperaceae | Areas of marshes and arctic vegetation, tropical areas of Asia, America and Africa. | Pollen | [52]; [59]; [60] |
| Cytinaceae | Mediterranean areas, Africa and Madagascar. Neotropics from Mexico to Colombia. | Pollen | [61]; [62] |
| Euphorbiaceae | Tropical zones and jungles, dry and hot deserts. | Pollen and nectar | [43]; [47]; [63] |
| Fabaceae | Andes mountain range, South America and its tropical areas | Pollen and nectar | [64]; [65] |
| Lardizabalaceae | Tropical forests, mixed temperate-warm forests of Asia and South America (eastern zone). | Pollen | [52]; [66]; [67] |
| Loranthaceae | Intertropical zones of South America, lowlands of neighboring areas of Chile and Argentina | Pollen and nectar | [68]; [69] |
| Lyrthaceae | Open forests, tropical and subtropical forests, rocky lands, grasslands, stream banks, mangroves, Asian and American coasts | Pollen | [52]; [70] |
| Melastomataceae | Tropical areas of Asia, America and Africa (pantropical). | Pollen and nectar | [43]; [71]; [72] |
| Molluginaceae | Tropical and subtropical regions of both hemispheres. Dry, open and hostile habitat | Pollen | [43]; [73] |
| Oleaceae | Temperate, cold and warm regions of Asia, Africa and South America. | Pollen and nectar | [52]; [74] |
| Papaveraceae | Central and western Asia, high mountains of Europe, America along the Pacific coast, in arid to warm climates | Pollen and nectar | [52]; [75]; [76] |
| Piperaceae | Tropical regions of the northern and southern hemispheres, with the highest concentration in Latin America and Malaysia, USA. | Pollen and nectar | [52]; [77]; [78] |
| Plantaginaceae | Temperate to tropical zones, considered a cosmopolitan family. | Pollen and nectar | [43]; [79]; [80] |
| Plumbaginaceae | Cold to arid and saline coastal habitats of Central Asia and subtropical and tropical regions of the Americas. | Pollen | [43]; [66] |
| Poaceae | From desert to humid environments, marine habitats and high mountains of tropical Asia, grasslands, pampas in South America and steppes of the old continent | Pollen | [81]; [82] |
| Polygalaceae | Tropical areas of America, Asia, Australia. | Pollen | [52]; [83]; [84] |
| Rosaceae | Wide distribution from cold to warm zones on five continents | Pollen and nectar | [43]; [85]; [126] |
| Salisaceae | Europe, Asia, Africa, all of America. | Pollen | [86]; [87]; [88] |
| Vitaceae | Tropical-humid regions of Asia and South America. | Pollen and nectar | [89]; [90]; [91] |
