# Supplementary material for: Pollen preferences of stingless bees in the Amazon region and southern highlands of Ecuador by scanning electron microscopy and morphometry
Source: PLoS One. 2022 Sep 20;17(9):e0272580. doi: 10.1371/journal.pone.0272580 (PMC9488792; doi:10.1371/journal.pone.0272580)
Supplement: S2 Table — (DOCX) [file pone.0272580.s002.docx]

**S2 Table. Morphological description, and their parameters, of pollen grains with the highest representation in the three study areas**

| **Pollen grain** | | **Taxonomic identification** | **Polar diameter**  **= P (µm)** | **Equatorial diameter**  **= E (µm)** | **Size** | **P/E** | **Shape** | **Exine** | **Ornamental Elements** |
| --- | --- | --- | --- | --- | --- | --- | --- | --- | --- |
| **Polar**  **view** | **Equatorial view** |  |  |  |  |  |  |  |  |
| 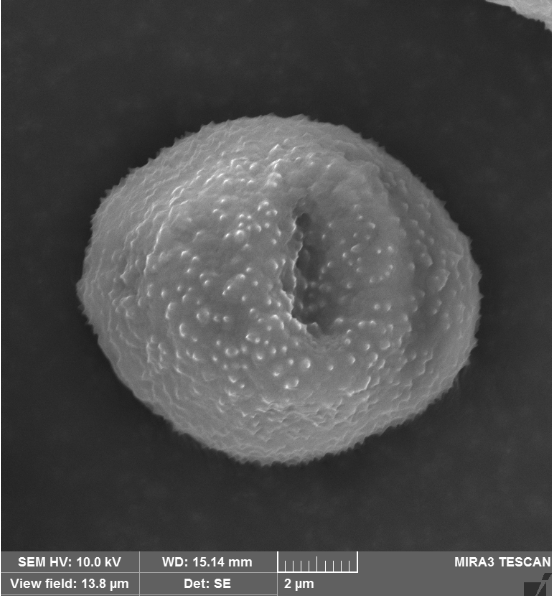 | 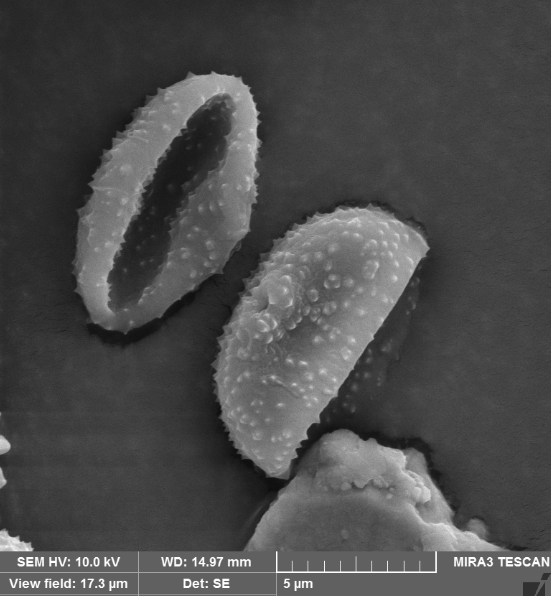 | Alismataceae | 8.64±2.08  Min: 5.31  Max: 13.93 | 8.35±1.80  Min: 5.88  Max: 11.73 | Tiny | 1.03 | Prolate-spheroidal | Microechinate | Microspinules and sulcus |
| 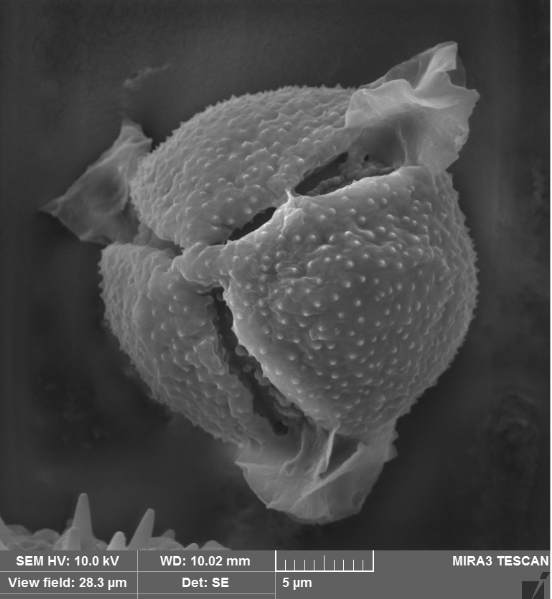 | 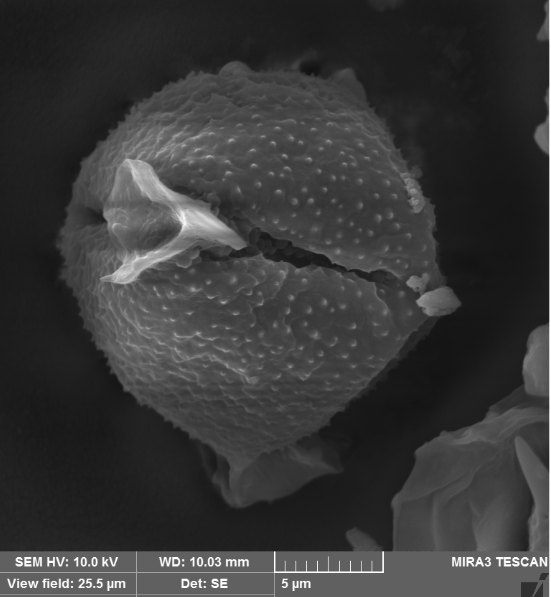 | Aizocaceae | 30.11±5.06  Min: 20.83  Max: 41.03 | 26.69±6.61  Min: 14.00  Max: 41.60 | Medium | 1.12 | Subprolate | Scabrate | Sulcus |
| 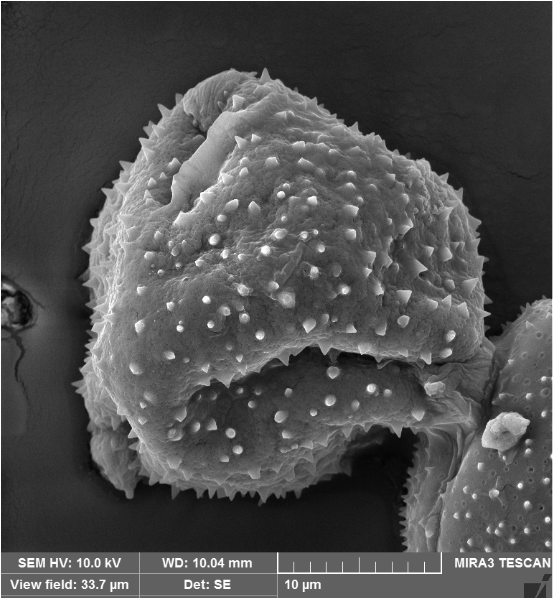 | 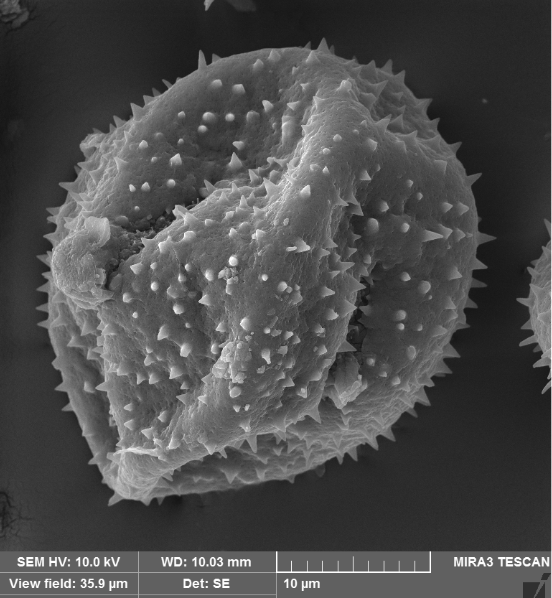 | Aizocaceae | 24.29±3.52  Min: 19.71  Max: 30.51 | 25.34±2.71  Min: 21.30  Max: 29.11 | Medium | 0.95 | Oval-spheroidal | Microechinate | Sulcus |
| 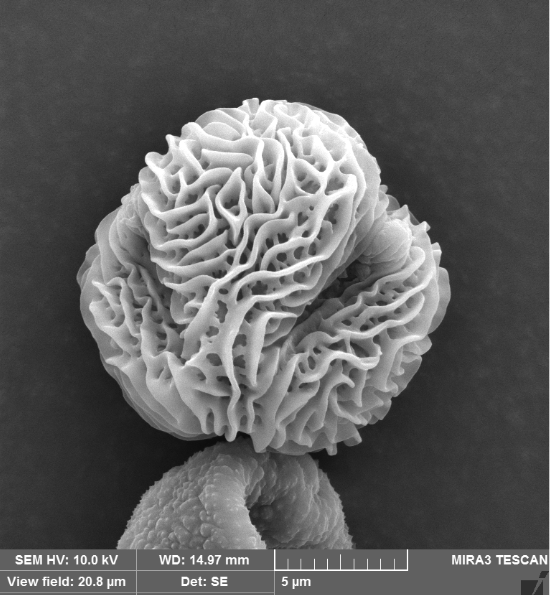 | 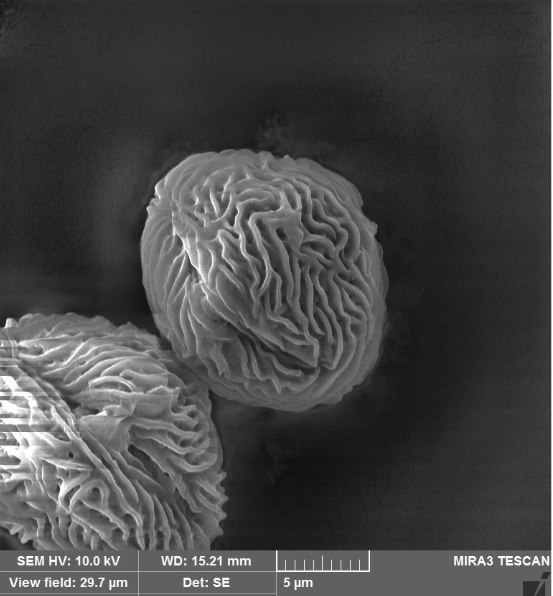 | Anacardaceae | 14.38±1.63  Min: 11.43  Max: 16.99 | 14.35±1.20  Min: 12.62  Max: 17.28 | Small | 1.00 | Spheroidal | Reticulate | High crests, pores and colpus |
| 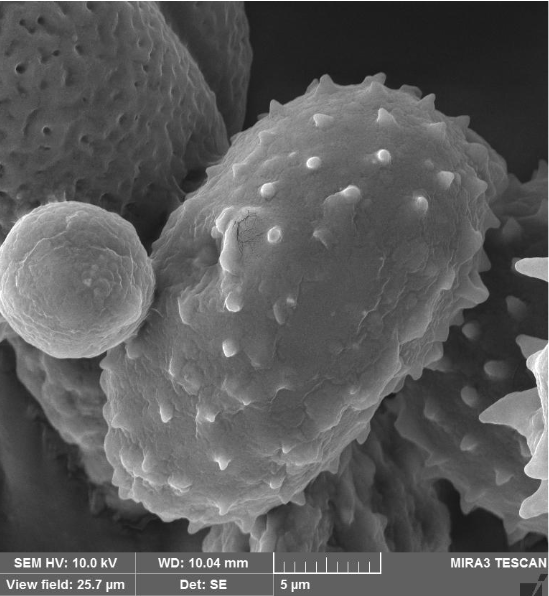 | 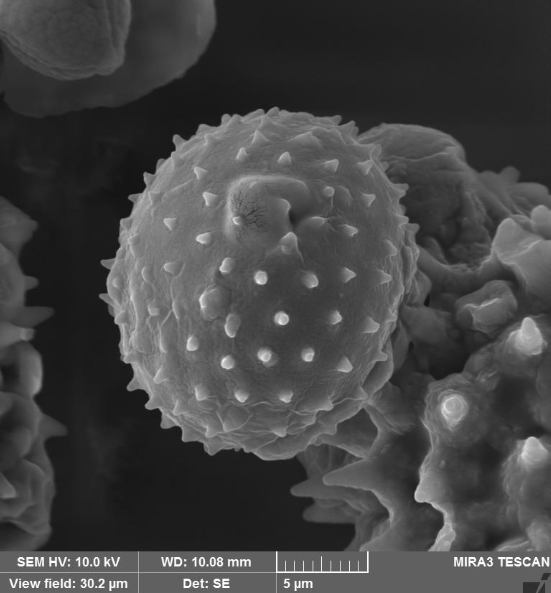 | Arecaceae | 21.40±3.18  Min: 17.41  Max: 26.42 | 19.12±1.94  Min: 16.74  Max: 22.03 | Small | 1.11 | Proladte-spheroidal | Microechinate | Pores |
| 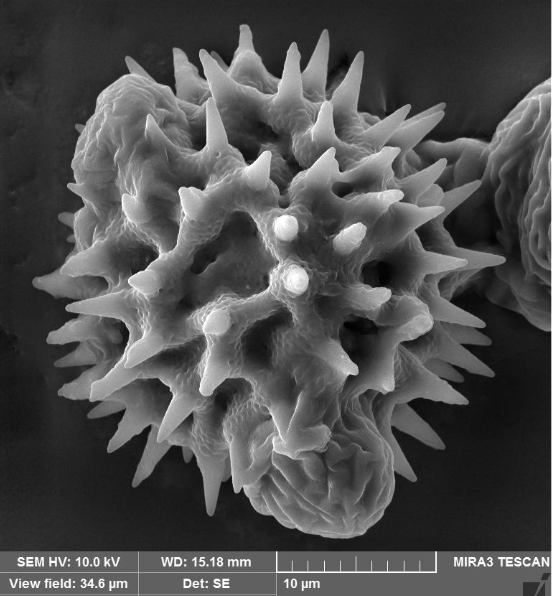 | 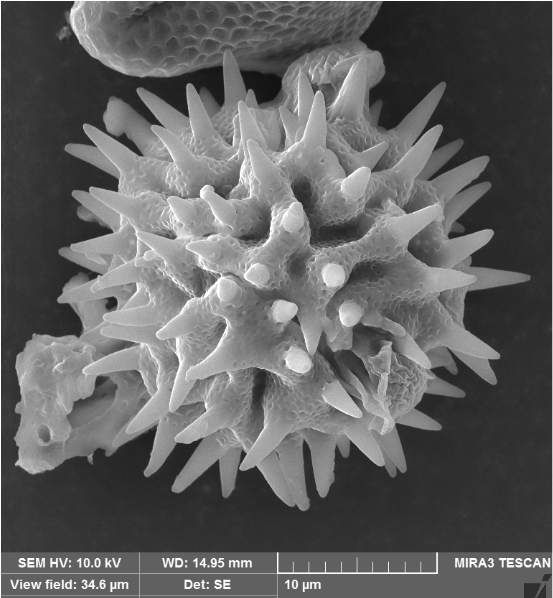 | Asteraceae  *Bidens*  [52] | 30.96±2.46  Min: 26.30  Max: 36.75 | 30.73±2.14  Min: 27.28  Max: 35.44 | Medium | 1.00 | Spheroidal | Echinate | Spikes and minipores |
| 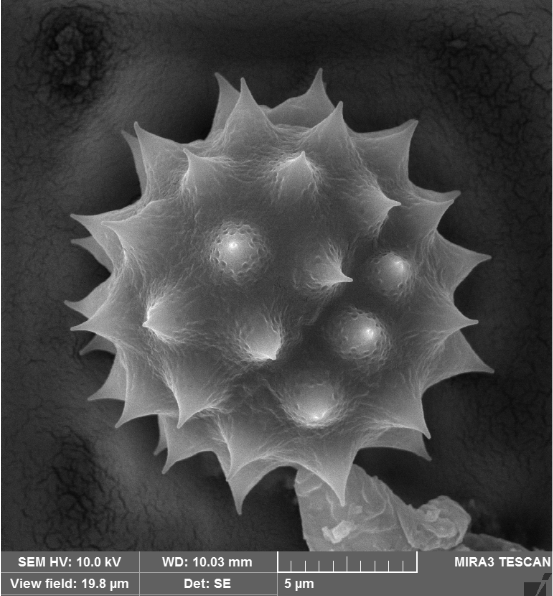 | 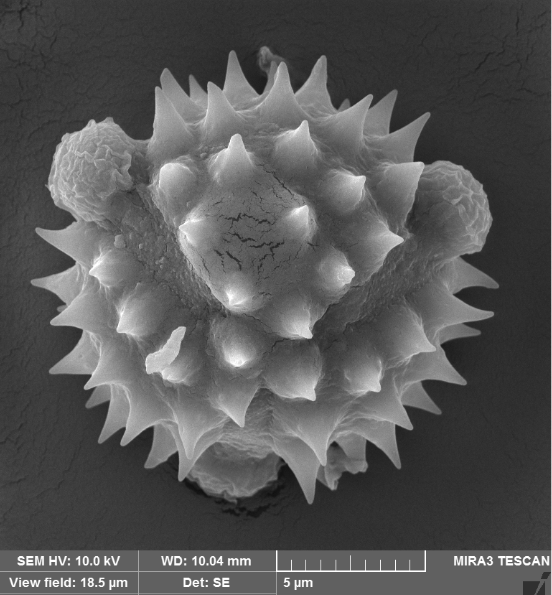 | Asteraceae  *Ageratum*  [52] | 16.53±1.38  Min: 12.98  Max: 19.83 | 16.62±1.74  Min: 14.34  Max: 20.81 | Small | 0.99 | Spheroidal | Echinate | Espinules and pores |
| 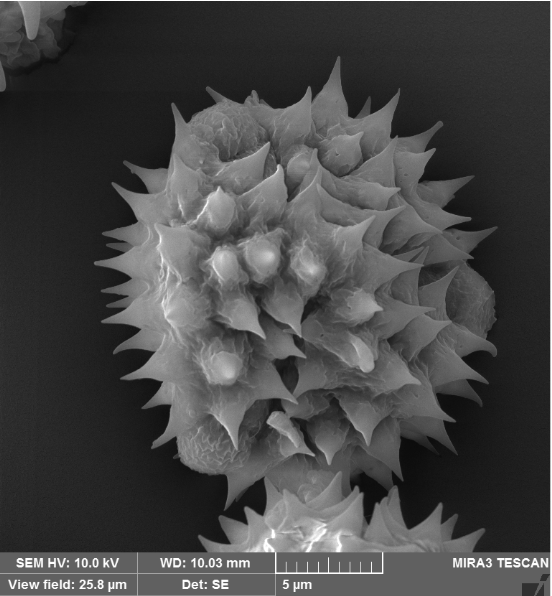 | 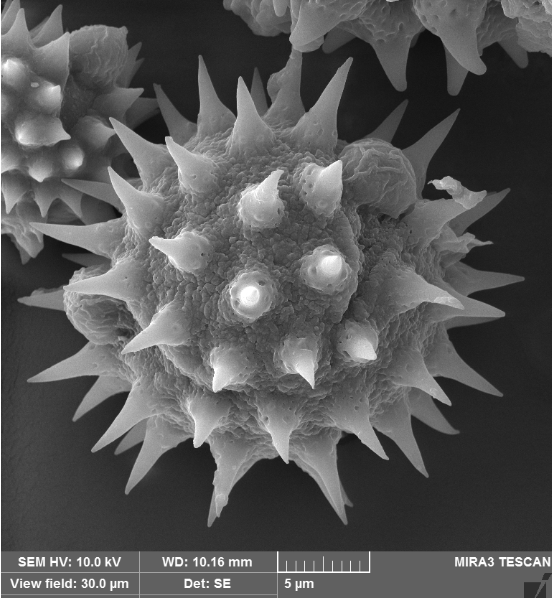 | Asteraceae  *Bidens*  [118] | 18.91±2.98  Min: 15.10  Max: 24.66 | 18.75±3.70  Min: 15.09  Max: 27.09 | Small | 1.00 | Spheroidal | Echinate | Spikes and pores |
| 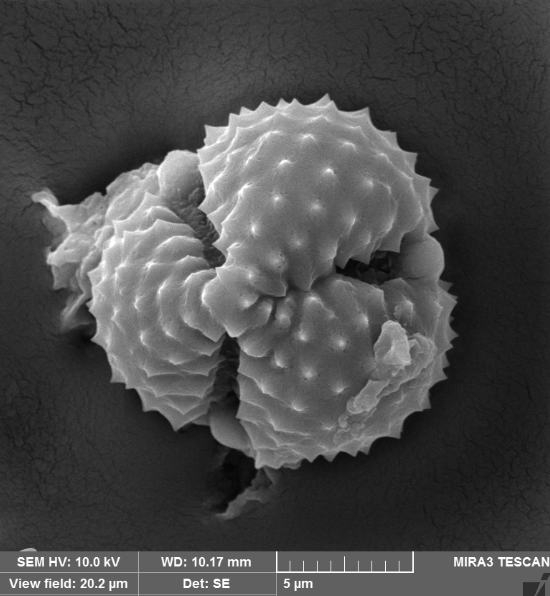 | 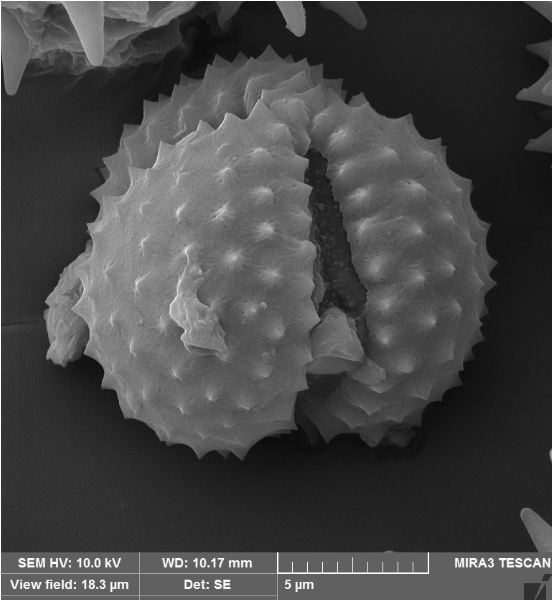 | Asteraceae  *Iva*  [52] | 13.68±0.75  Min: 12.58  Max: 15.65 | 13.56±0.97  Min: 11.73  Max: 15.1 | Small | 1.00 | Spheroidal | Microechinate | Microespinules and colpus |
| 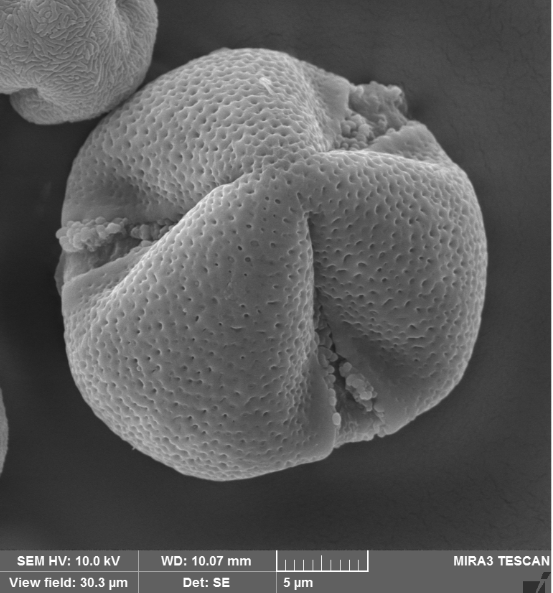 | 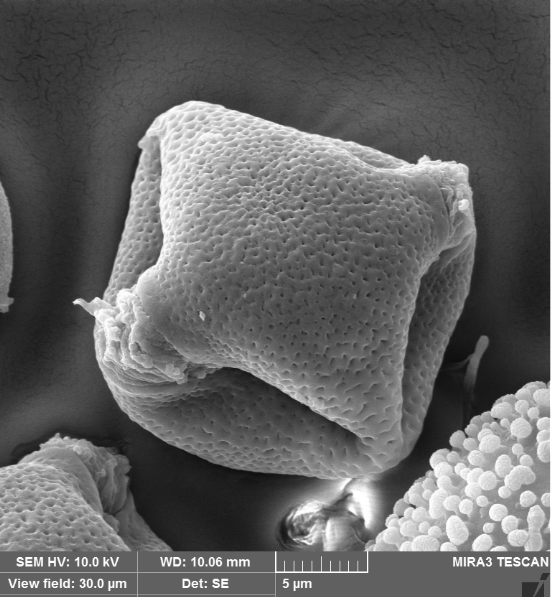 | Berberidaceae | 22.79±1.80  Min: 18.13  Max: 25.23 | 23.39±2.89  Min: 18.95  Max: 30.32 | Medium | 0.97 | Oval-spheroidal | Miniporate | Colpus y pores |
| 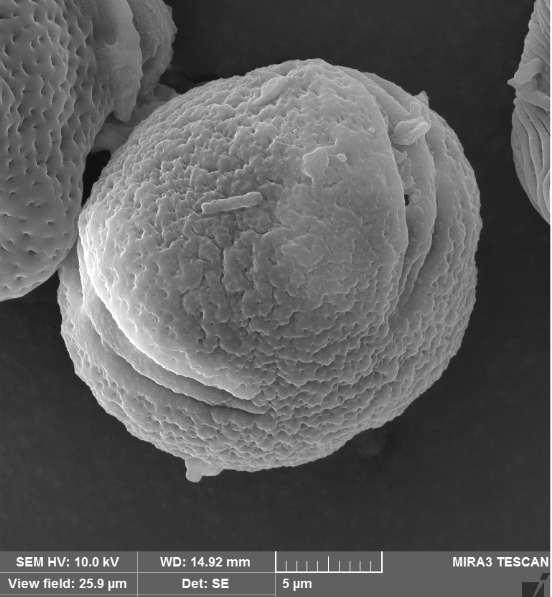 | 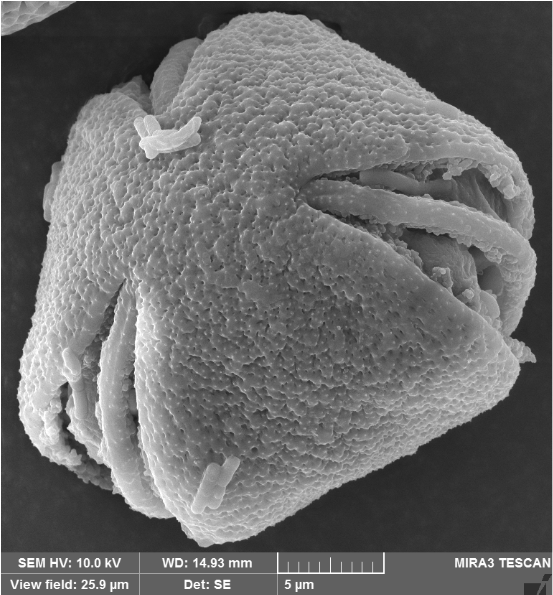 | Bursecaceae  *Bursera*  [43] | 20.30±0.58  Min: 19.80  Max: 21.04 | 18.05±3.09  Min: 13.78  Max: 21.56 | Small | 1.12 | Prolate-spheroidal | Granulate | Sulcus and ribs |
| 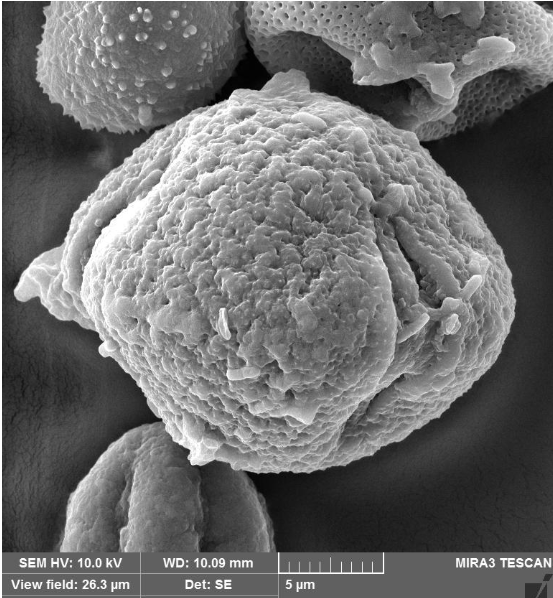 | 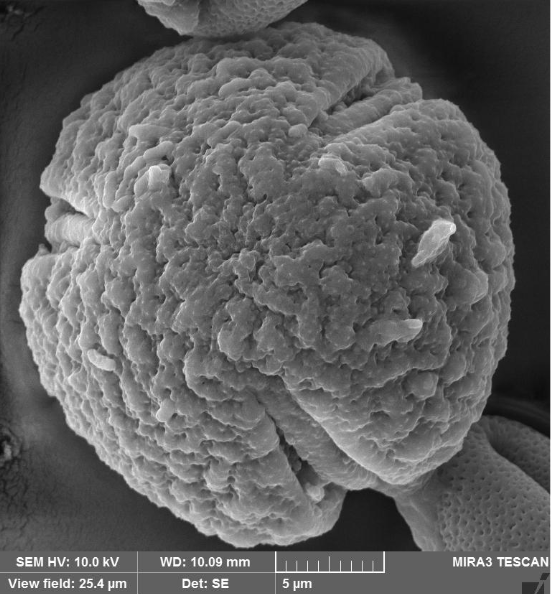 | Bursecaceae  *Quercus*  [119] | 20.34±1.35  Min: 17.98  Max: 21.71 | 22.268±1.75  Min: 19.39  Max: 24.18 | Small | 0.91 | Oval-spheroidal | Verrucate | Sulcus and ribs |
| 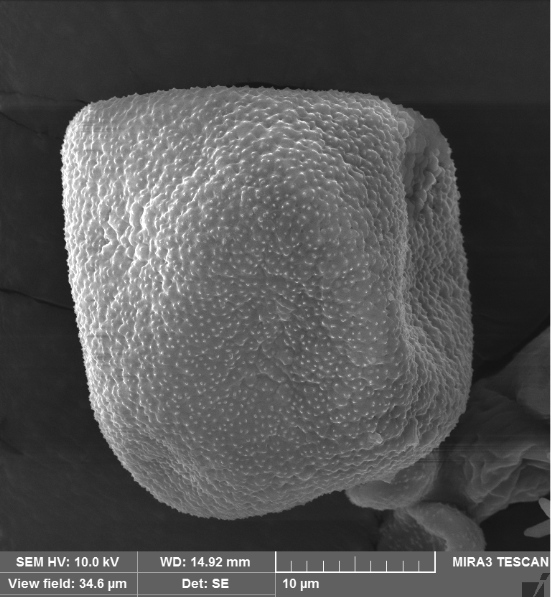 | 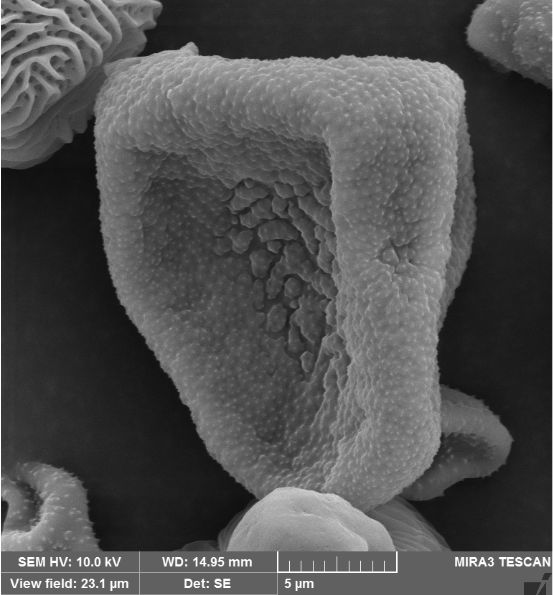 | Cyperaceae  *Cyperus*  [120,121] | 19.73±3.50  Min: 12.44  Max: 27.97 | 19.28±3.03  Min: 15.33  Max: 27.32 | Small | 1.02 | Prolate-spheroidal | Granulate | Pseudosulcus |
| 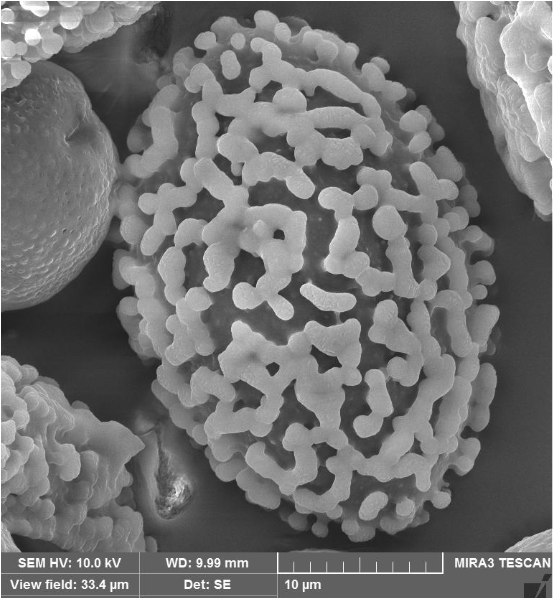 | 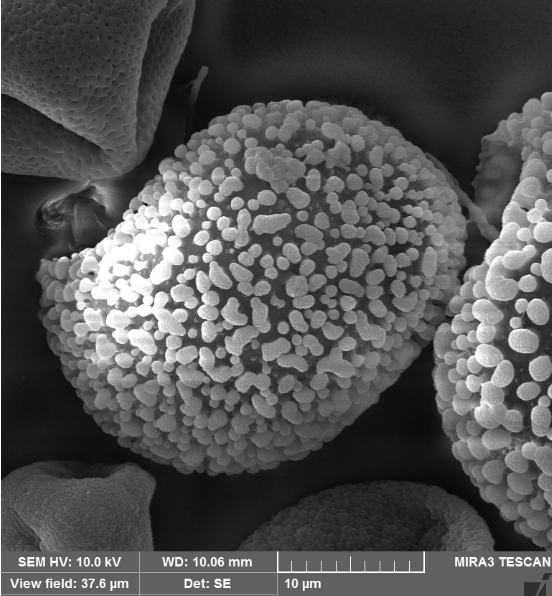 | Cytinaceae | 28.18±2.33  Min: 23.75  Max: 32.64 | 27.16±3.51  Min: 21.28  Max: 31.73 | Medium | 1.03 | Spheroidal | Gemmate | Sulcus |
| 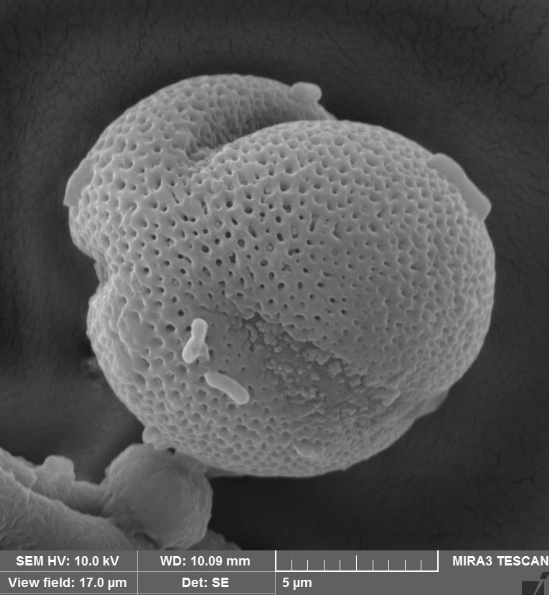 | 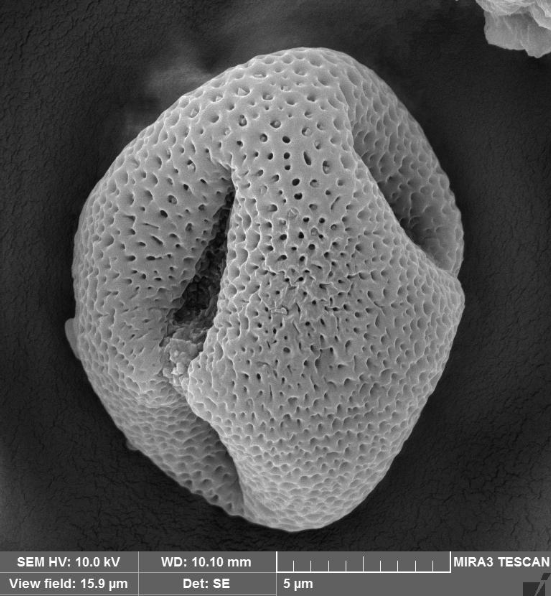 | Euphorbiaceae | 15.80±4.48  Min: 10.69  Max: 22.89 | 14.75±2.81  Min: 9.19  Max: 22.80 | Small | 1.07 | Prolate-spheroidal | Porate, reticulate | Colpus |
| 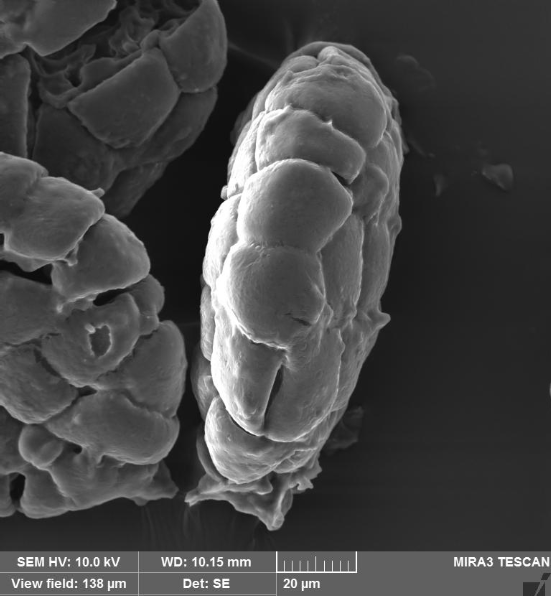 | 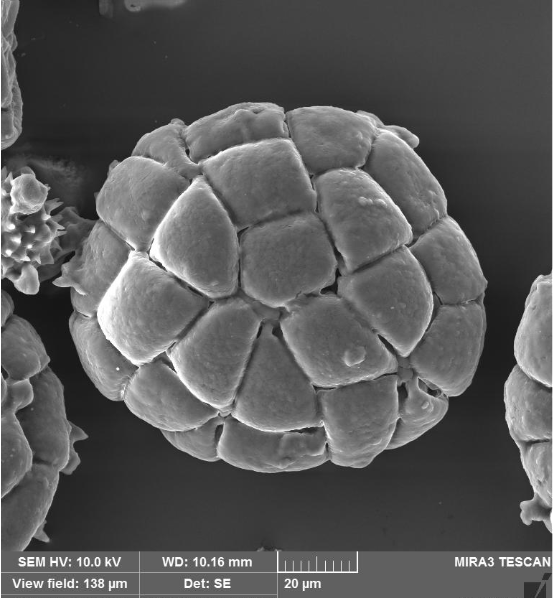 | Fabaceae  *Inga*  [122] | 92.43±21.51  Min: 54.68  Max: 120.69 | 104.31±10.89  Min: 92.00  Max: 125.48 | Big | 0.88 | Oval-spheroidal | Psilate | Polyad (21-24 pollen grains) |
| 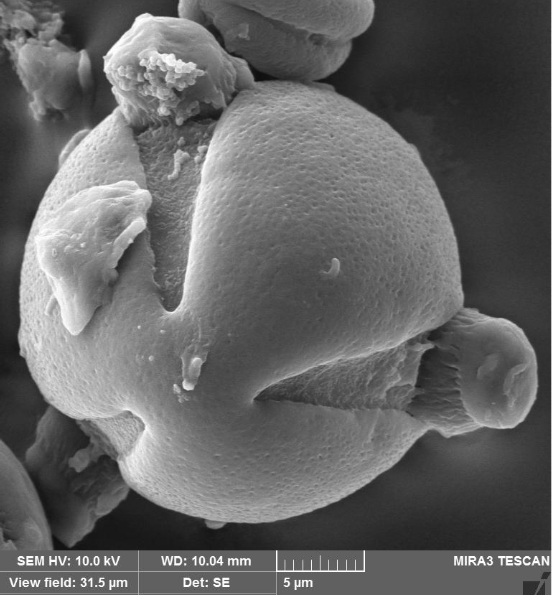 | 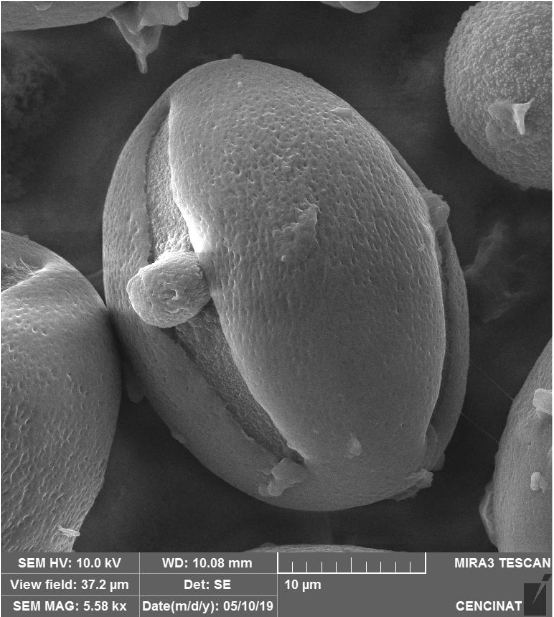 | Fabaceae  Chamaecrista  [123] | 25.28±4.02  Min: 17.78  Max: 30.35 | 28.20±4.26  Min: 17.82  Max: 34.41 | Medium | 0.89 | Oval-spheroidal | Microperforate | Colpus and pores |
| 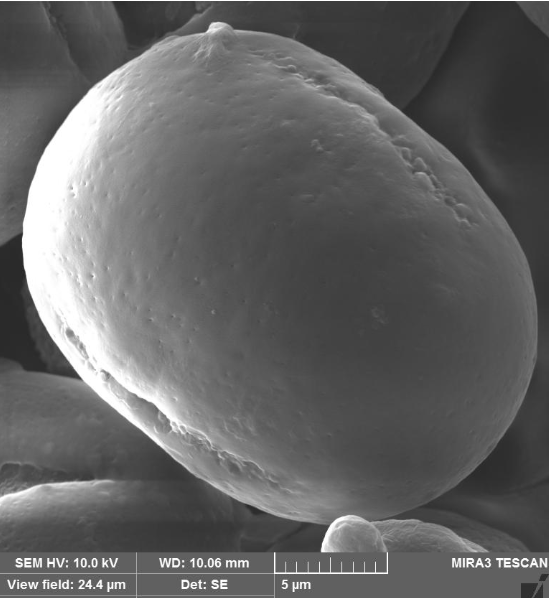 |  | Fabaceae | 18.82 | 21.29 | Small | 0.88 | Oval-spheroidal | Psilate | Sulcus |
| 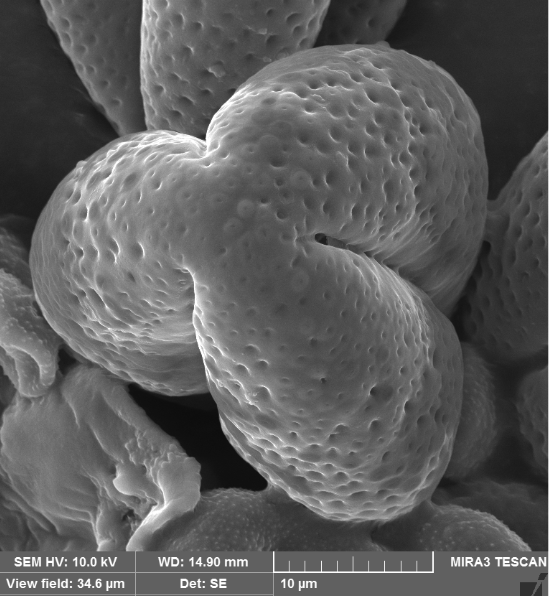 | 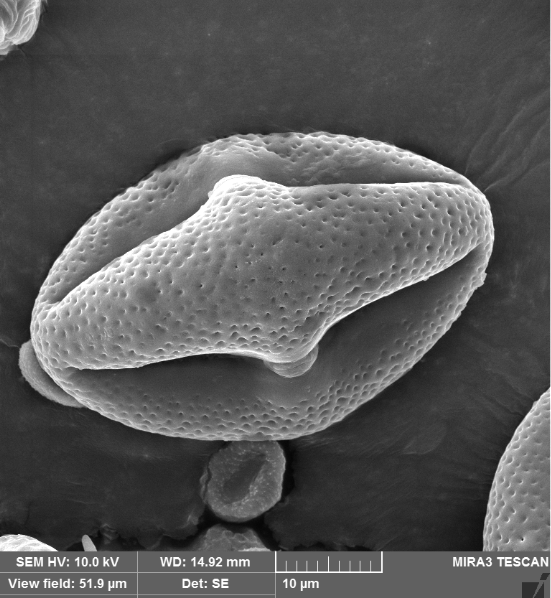 | Fabaceae | 29.90±0.99  Min: 29.19  Max: 30.60 | 36.46±10.91  Min: 28.479  Max: 44.18 | Medium | 0.81 | Sub-oval | Microperforate | Colpus |
| 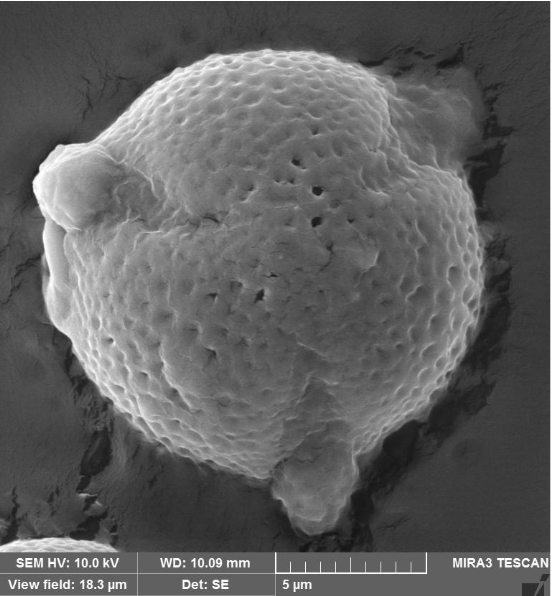 | 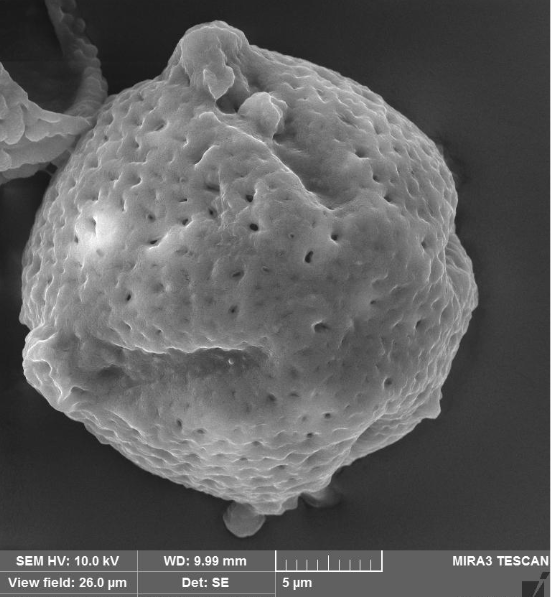 | Lardizabalaceae | 17.23±3.69  Min: 12.07  Max: 25.10 | 15.51±3.35  Min: 11.94  Max: 24.76 | Small | 1.11 | Prolate-speroidal | Microreticulate | Colpus and pores |
| 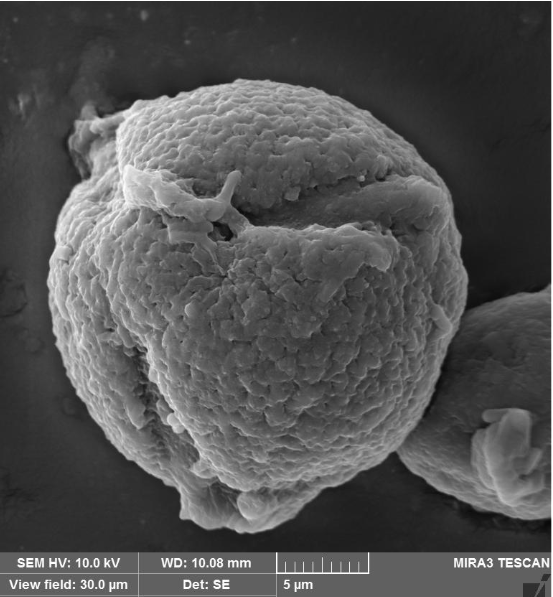 | 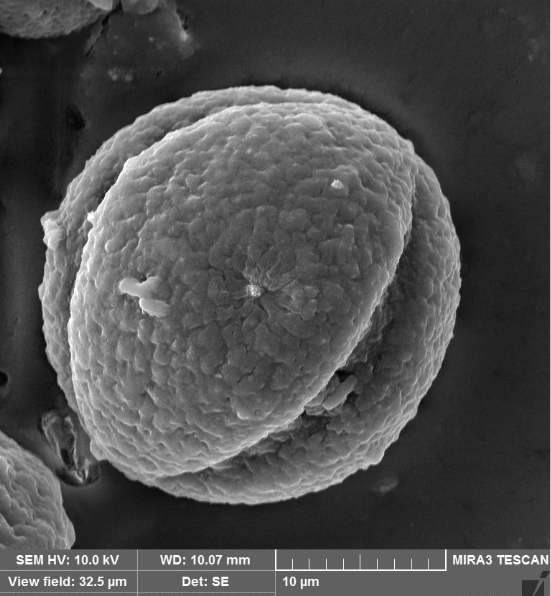 | Lythraceae  *Hemia*  [124] | 23.46±2.43  Min: 17.83  Max: 30.05 | 23.37±1.87  Min: 19.13  Max: 28.63 | Medium | 1.00 | Spheroidal | Scabrate | Colpus and pores |
| 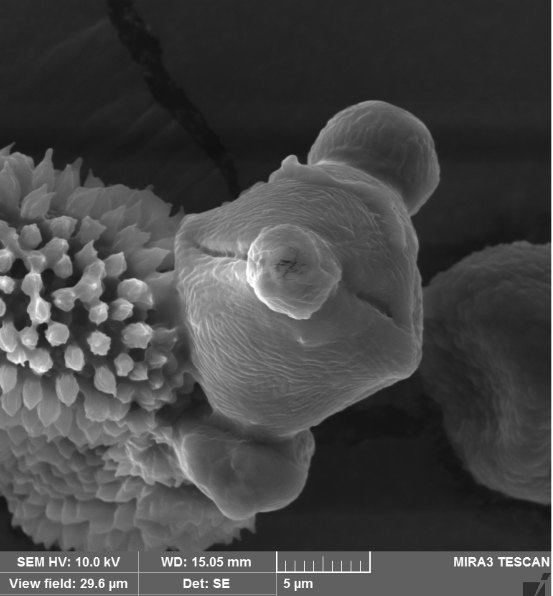 | 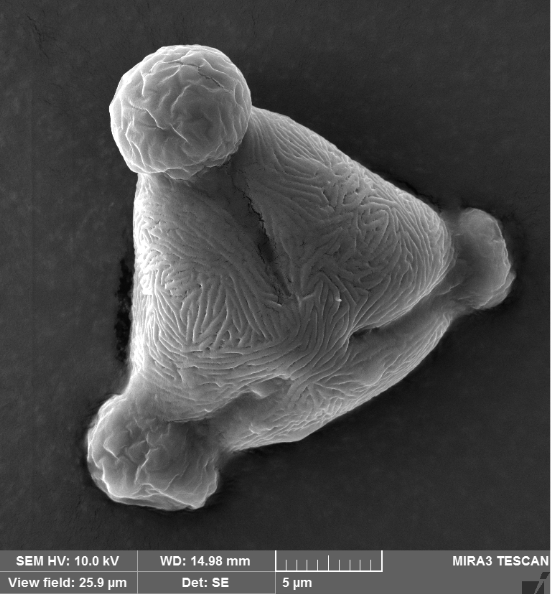 | Loranthaceae | 16.98±3.54  Min: 12.41  Max: 22.21 | 20.33±1.53  Min: 18.28  Max: 21.97 | Small | 0.83 | Sub-oval | Fossulate | Sulcus and pores |
| 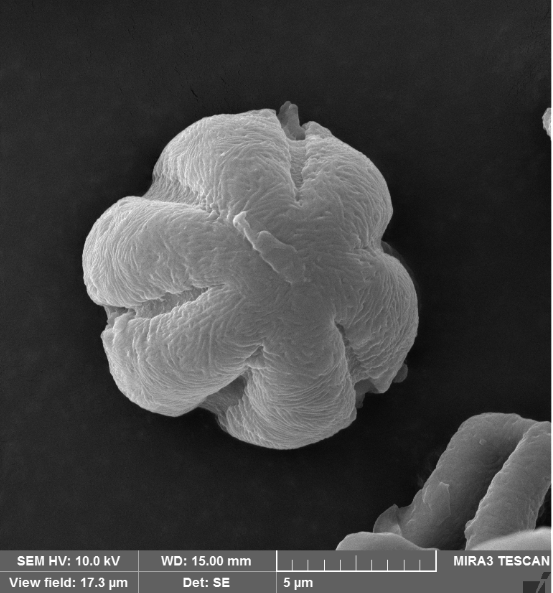 | 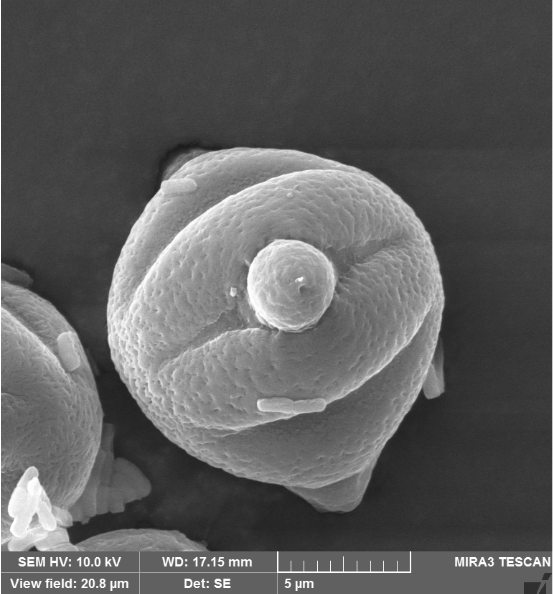 | Melastomataceae  *Miconia*  [43, 99] | 12.17±1.92  Min: 8.61  Max: 5.03 | 12.65±1.39  Min: 10.01  Max: 6.16 | Small | 0.96 | Oval-spheroidal | Scabrate | Colpus and pores |
| 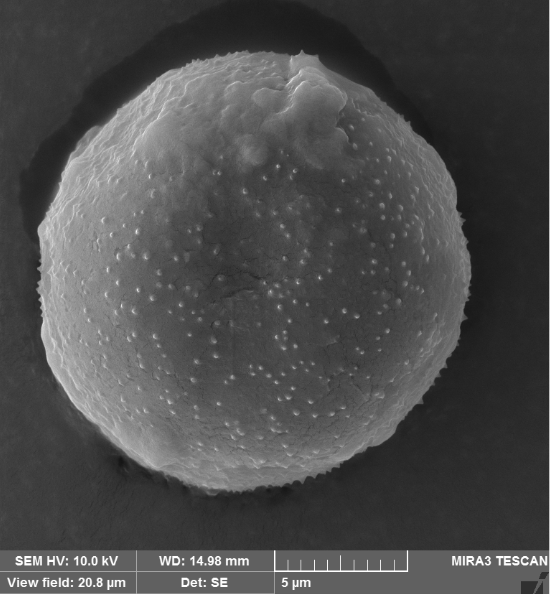 | 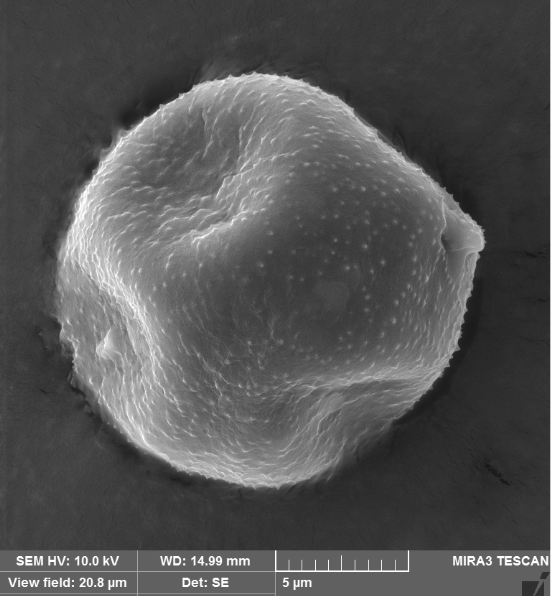 | Molluginaceae  *Mollugo*  [43,47] | 15.97±0.41  Min: 15.68  Max: 16.27 | 16.01±1.29  Min: 14.09  Max: 16.91 | Small | 0.99 | Oval-spheroidal | Echinate | Pseudosulcus |
| 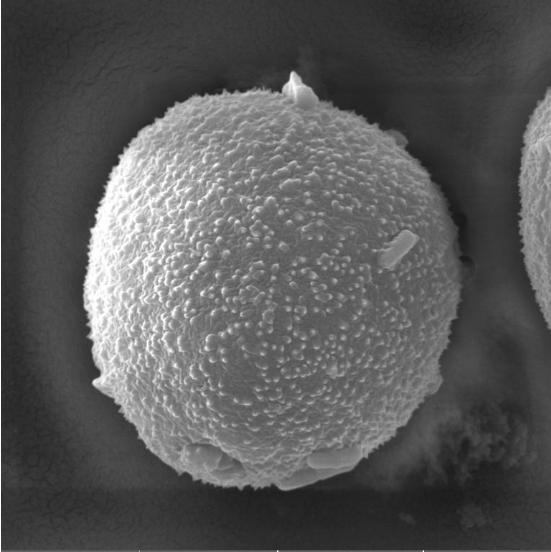 | 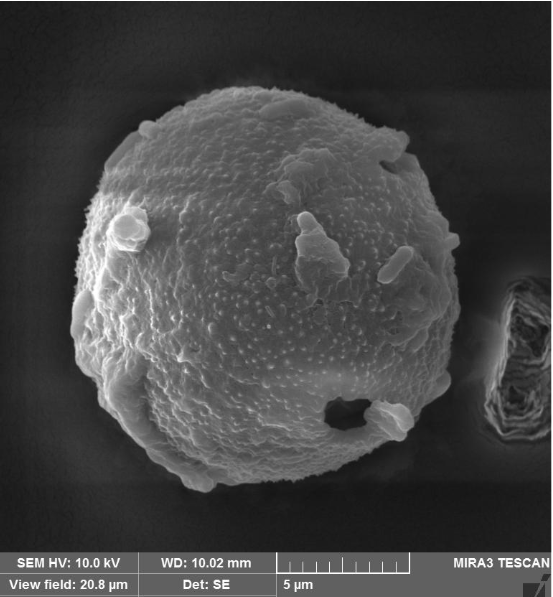 | Molluginaceae | 15.45±2.09  Min: 13.11  Max: 22.15 | 14.84±13.10  Min: 13.10  Max: 16.39 | Small | 1 | Spheroidal | Scabrate | Pores |
| 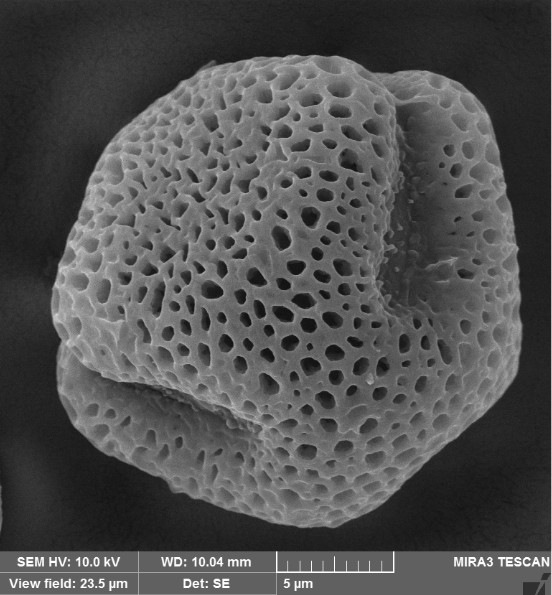 |  | Oleaceae  *Fraxinus*  [100] | 20.21 | NI | Small | NI | NI | Porate | Sulcus |
| 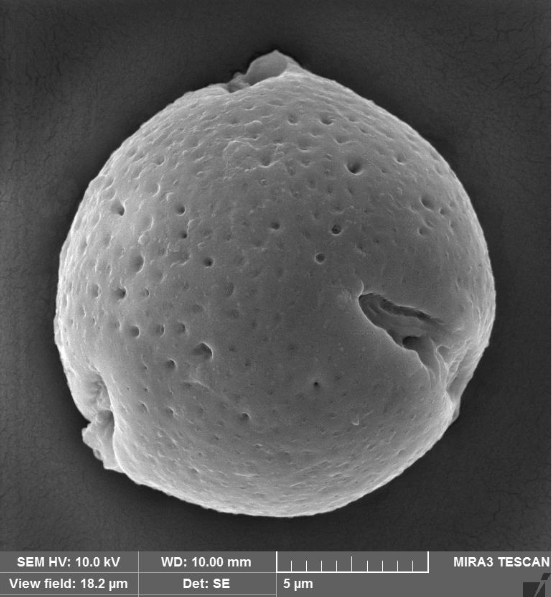 | 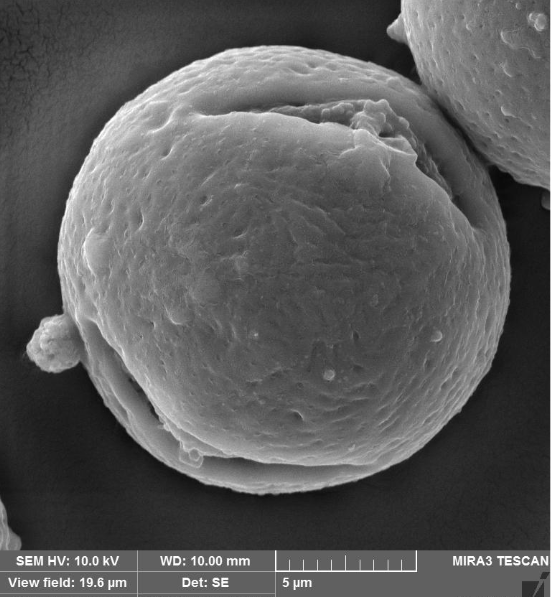 | Papaveraceae | 16.95±2.11  Min: 14.82  Max: 20.63 | 15.53±1.53  Min: 13.05  Max: 17.81 | Small | 1.09 | Prolate-spheroidal | Psilate | Sulcus |
| 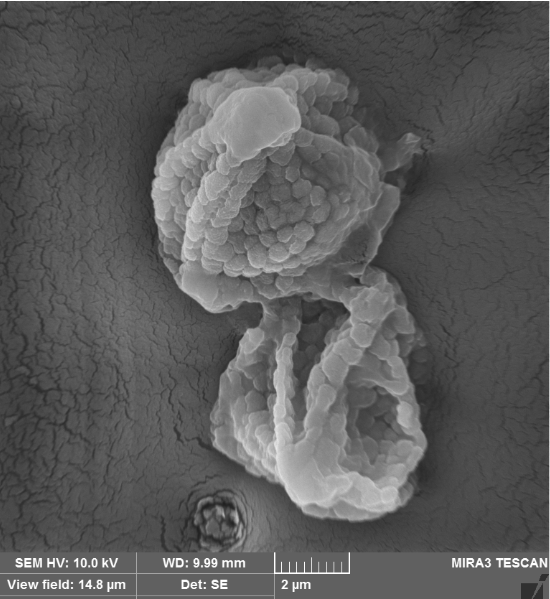 |  | Piperaceae | 7.17±1.17  Min: 5.83  Max: 11.68 | 6.66±0.50  Min: 5.52  Max: 7.40 | Tiny | 1.07 | Prolate-spheroidal | Granulate | Colpus |
| 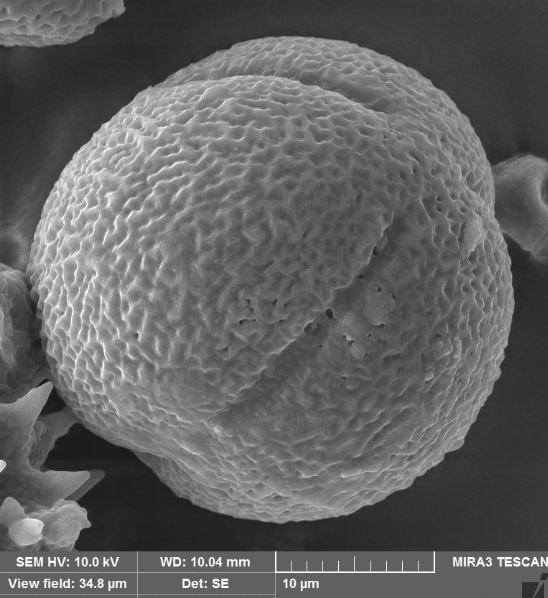 | 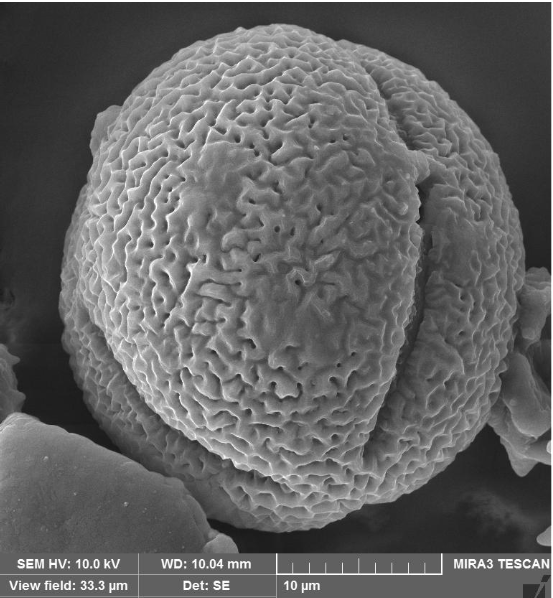 | Plantaginaceae | 28.33±2.72  Min: 22.52  Max: 34.59 | 29.20±3.62  Min: 22.11  Max: 35.50 | Medium | 0.97 | Oval-spheroidal | Rugulate | Colpus |
| 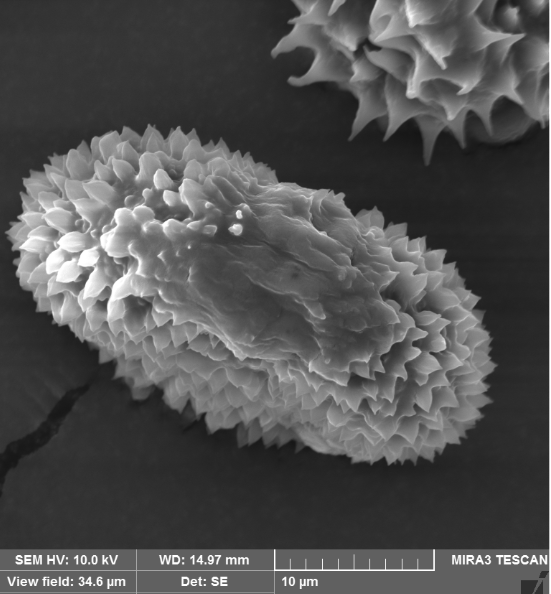 | 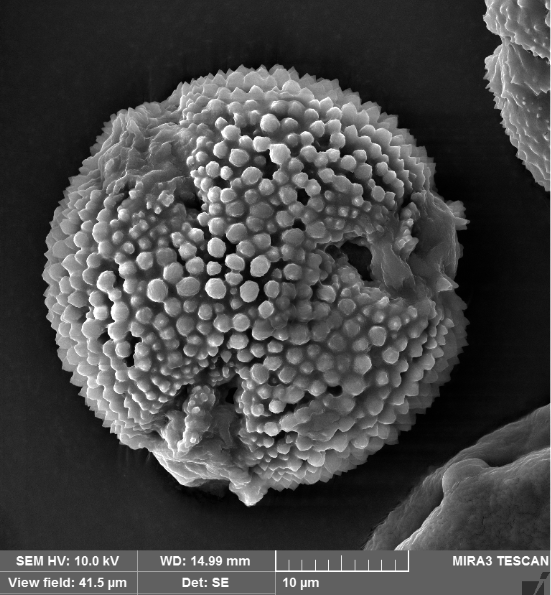 | Plumbaginaceae  *Plumbago*  [43,125] | 26.19±6.74  Min: 21, 42  Max: 30.96 | 33.27±0.56  Min: 32.56  Max: 33.79 | Medium | 0.78 | Sub-oval | Pilate | Mesocolpus or sulcus |
| 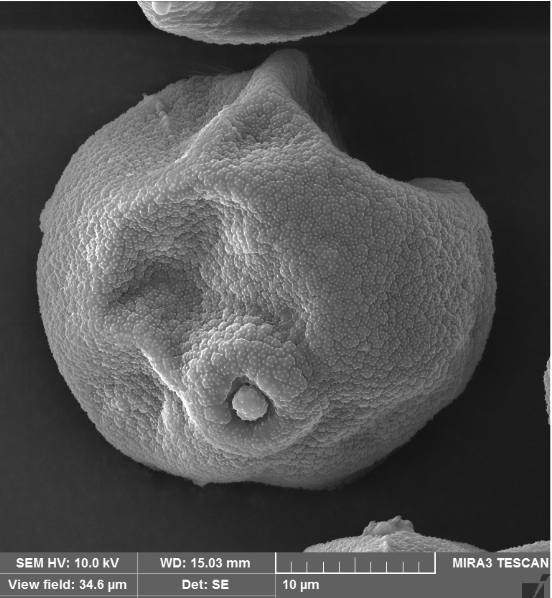 | 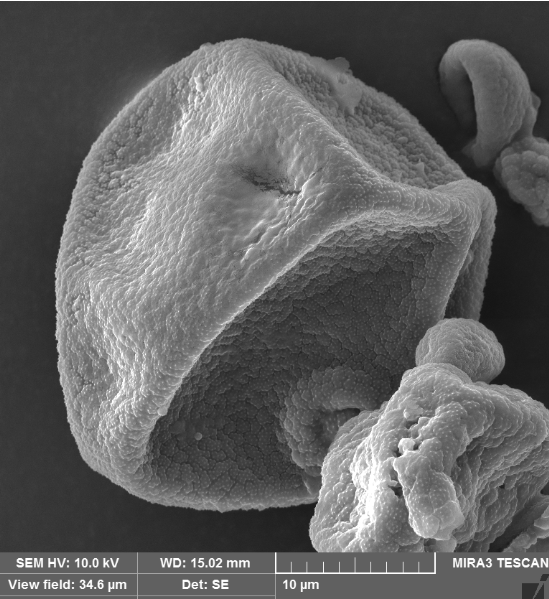 | Poaceae | 36.63±7.36  Min: 27.54  Max: 53.07 | 36.88±6.09  Min: 27.45  Max: 45.70 | Medium | 0.99 | Oval-spheroidal | Granulate | Pores |
| 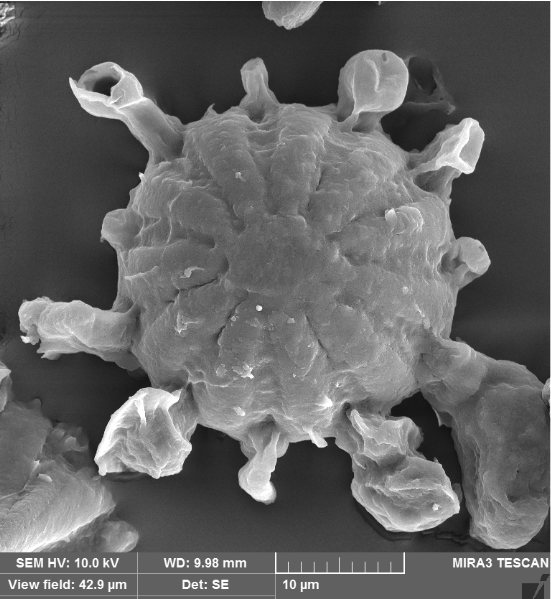 | 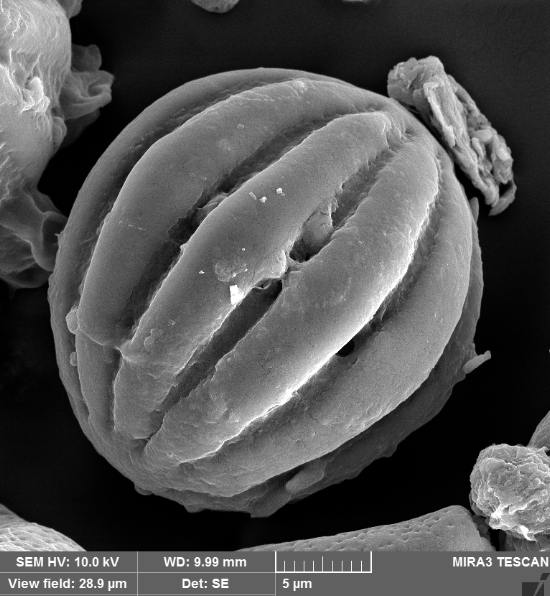 | Polygalaceae  *Polygala*  [84] | 30.74±3.45  Min: 25.69  Max: 37.7 | 28.66±3.98  Min: 22.64  Max: 34.92 | Medium | 1.07 | Prolate-spheroidal | Psilate | Colpus |
| 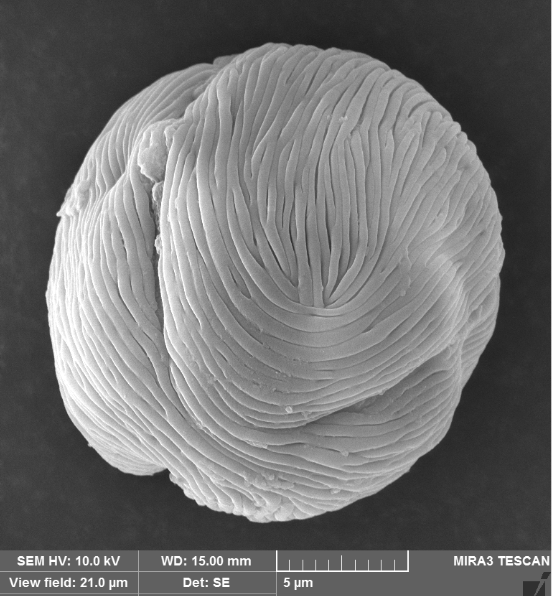 | 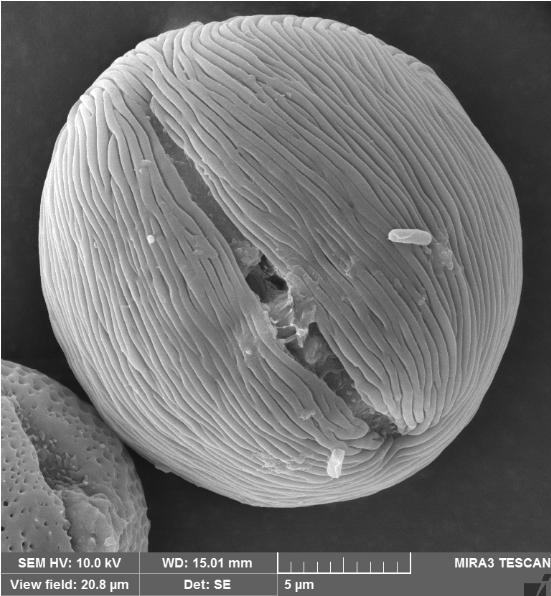 | Rosaceae  *Prunus*  [86,126] | 17.39±0.03  Min: 17.37  Max: 17.42 | 18.27±3.51  Min: 13.17  Max: 23.36 | Small | 0.95 | Oval - spheroidal | Striate | Colpus and pores |
| 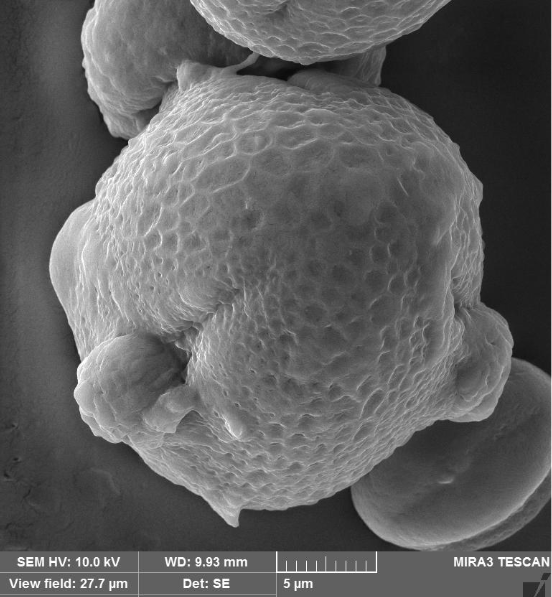 | 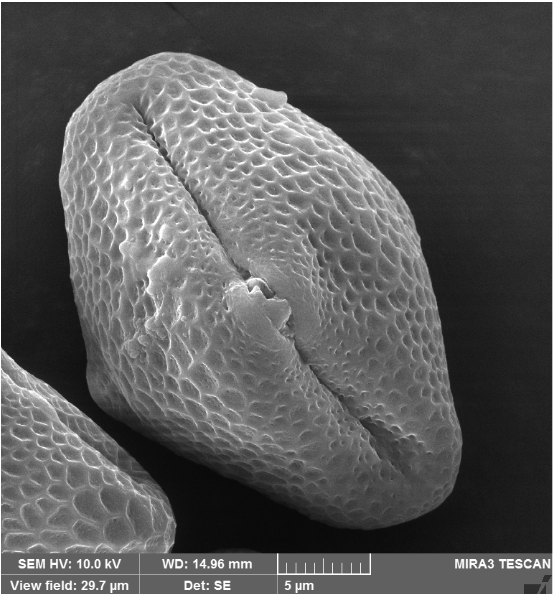 | Salisaceae  *Salix*  [127] | 25.27±2.74  Min: 19.85  Max: 29.89 | 24.21±4.13  Min: 17.13  Max: 35.41 | Medium | 1.04 | Spheroidal | Faveolate | Colpus and pores |
| 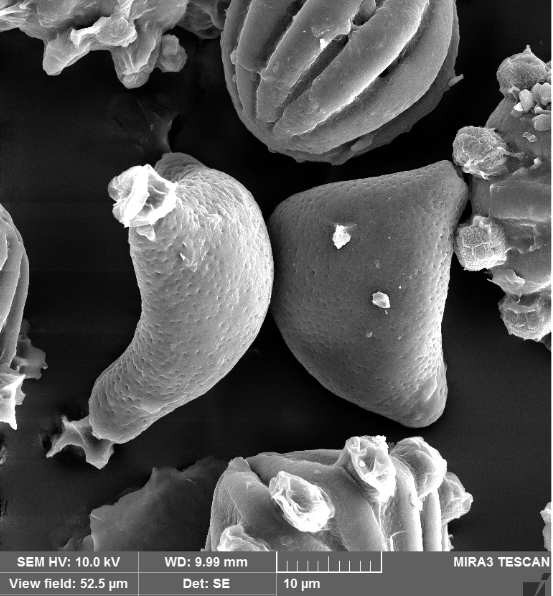 |  | Sapindaceae  *Paullinia*  [128] | 25.83±4.94  Min: 15.51  Max: 36.80 | 30.56±4.95  Min: 18.57  Max: 42.25 | Medium | 0.84 | Sub - oval | Microperforated | Sulcus and pores |
| 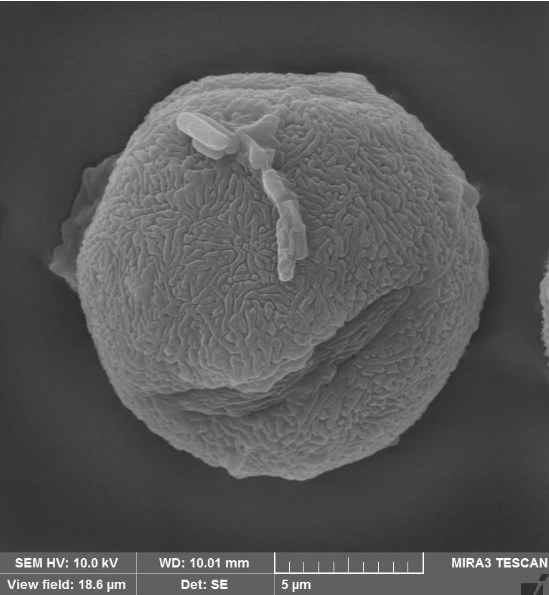 | 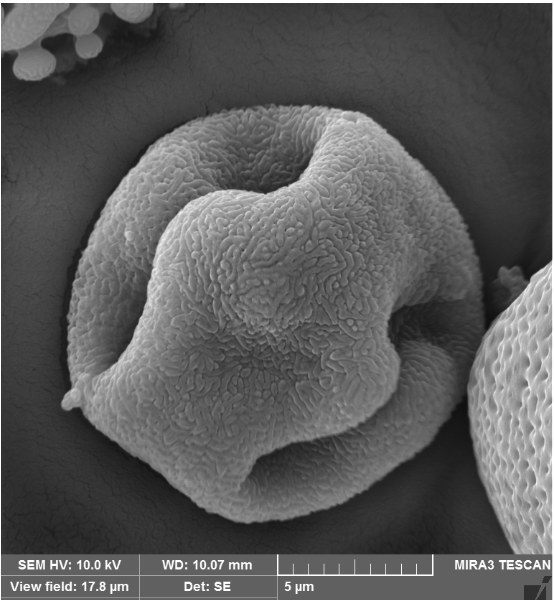 | Vitaceae  *Cissus*  [129] | 14.03±1.26  Min: 11.06  Max: 16.02 | 15.97±5.72  Min: 11.74  Max:32.22 | Small | 0.87 | Oval - spheroidal | Fossulate | Sulcus |
