# Supplementary material for: Pollen preferences of stingless bees in the Amazon region and southern highlands of Ecuador by scanning electron microscopy and morphometry
Source: PLoS One. 2022 Sep 20;17(9):e0272580. doi: 10.1371/journal.pone.0272580 (PMC9488792; doi:10.1371/journal.pone.0272580)
Supplement: S3 Table — Comparison between the diversity, dominance and similarity indices in each study area. (DOCX) [file pone.0272580.s003.docx]

**S3 Table. Indexes explaining chart.** Comparison between the diversity, dominance and similarity indices in each study area.

| Region | Province | Locality | Nest | Diversity | | | | Dominance Simpson | Similarity Jaccard |
| --- | --- | --- | --- | --- | --- | --- | --- | --- | --- |
|  |  |  |  | **Shannon Mean** | **Alpha Mean** | **Chao 1 Mean** | **Chao 2 Mean** |  |  |
| Amazonian | Sucumbios | Shushufindi | H1N1 | 0.97 | 0.51 | 4.21 | 4.21 | 0.39 | 0.436 |
|  |  |  | H2N1 | 1.45 | 0.96 | 8.13 | 22.56 | 0.27 | 0.136 |
|  |  |  | H3N2 | 1.72 | 1.26 | 11.04 | 34.47 | 0.23 | 0.146 |
|  | Orellana | Dayuma | H1N2 | 1.86 | 1.51 | 13.31 | 39.4 | 0.22 | 0.495 |
|  |  | Justicia Social | H2N1 | 2.01 | 1.81 | 16.16 | 43.87 | 0.20 | 0.297 |
|  |  | 24 de agosto | H3N1 | 2.11 | 2.02 | 18.18 | 46.8 | 0.19 | 0.267 |
|  |  | La Merced | H4N2 | 2.17 | 2.18 | 19.7 | 45.96 | 0.18 | 0.189 |
| Sierra | Loja | Cristo del Conzuelo | H1N5 | 2.22 | 2.34 | 21.3 | 51.09 | 0.17 | 0.387 |
|  |  | Naranjito | H2N1 | 2.26 | 2.49 | 22.81 | 48.15 | 0.17 | 0.375 |
|  |  | El Toro | H3N3 | 2.29 | 2.63 | 24.3 | 46.21 | 0.17 | 0.235 |
|  |  | 12 de Octubre | H5N1 | 2.31 | 2.77 | 25.76 | 45.84 | 0.17 | 0.429 |
|  |  | Curiachi | H6N1 | 2.32 | 2.87 | 26.82 | 48.5 | 0.17 | 0.571 |
|  |  | 12 de Diciembre | H7N1 | 2.34 | 2.98 | 28 | 47.69 | 0.17 | 0.645 |
